# A New 4D Trajectory-Based Approach Unveils Abnormal LV Revolution Dynamics in Hypertrophic Cardiomyopathy

**DOI:** 10.1371/journal.pone.0122376

**Published:** 2015-04-13

**Authors:** Andrea Madeo, Paolo Piras, Federica Re, Stefano Gabriele, Paola Nardinocchi, Luciano Teresi, Concetta Torromeo, Claudia Chialastri, Michele Schiariti, Geltrude Giura, Antonietta Evangelista, Tania Dominici, Valerio Varano, Elisabetta Zachara, Paolo Emilio Puddu

**Affiliations:** 1 Dipartimento di Scienze Cardiovascolari, Respiratorie, Nefrologiche, Anestesiologiche e Geriatriche, Sapienza—Università di Roma, Rome, Italy; 2 Centro per le Cardiomiopatie Ospedale S. Camillo-Forlanini, Roma, Italy; 3 Dipartimento di Scienze, Università Roma Tre, Rome, Italy; 4 Center for Evolutionary Ecology, Rome, Italy; 5 Dipartimento di Ingegneria Strutturale e Geotecnica, Sapienza—Università di Roma, Rome, Italy; 6 Dipartimento di Architettura, LaMS—Modeling & Simulation Lab, Università Roma Tre, Rome, Italy; 7 Dipartimento di Matematica e Fisica, LaMS—Modeling & Simulation Lab, Università Roma Tre, Rome, Italy; 8 Ospedale San Giovanni Calibita Fatebenefratelli—Isola Tiberina, Rome, Italy; Gent University, BELGIUM

## Abstract

The assessment of left ventricular shape changes during cardiac revolution may be a new step in clinical cardiology to ease early diagnosis and treatment. To quantify these changes, only point registration was adopted and neither Generalized Procrustes Analysis nor Principal Component Analysis were applied as we did previously to study a group of healthy subjects. Here, we extend to patients affected by hypertrophic cardiomyopathy the original approach and preliminarily include genotype positive/phenotype negative individuals to explore the potential that incumbent pathology might also be detected. Using 3D Speckle Tracking Echocardiography, we recorded left ventricular shape of 48 healthy subjects, 24 patients affected by hypertrophic cardiomyopathy and 3 genotype positive/phenotype negative individuals. We then applied Generalized Procrustes Analysis and Principal Component Analysis and inter-individual differences were cleaned by Parallel Transport performed on the tangent space, along the horizontal geodesic, between the per-subject consensuses and the grand mean. Endocardial and epicardial layers were evaluated separately, different from many ecocardiographic applications. Under a common Principal Component Analysis, we then evaluated left ventricle morphological changes (at both layers) explained by first Principal Component scores. Trajectories’ shape and orientation were investigated and contrasted. Logistic regression and Receiver Operating Characteristic curves were used to compare these morphometric indicators with traditional 3D Speckle Tracking Echocardiography global parameters. Geometric morphometrics indicators performed better than 3D Speckle Tracking Echocardiography global parameters in recognizing pathology both in systole and diastole. Genotype positive/phenotype negative individuals clustered with patients affected by hypertrophic cardiomyopathy during diastole, suggesting that incumbent pathology may indeed be foreseen by these methods. Left ventricle deformation in patients affected by hypertrophic cardiomyopathy compared to healthy subjects may be assessed by modern shape analysis better than by traditional 3D Speckle Tracking Echocardiography global parameters. Hypertrophic cardiomyopathy pathophysiology was unveiled in a new manner whereby also diastolic phase abnormalities are evident which is more difficult to investigate by traditional ecocardiographic techniques.

## Introduction

Hypertrophic cardiomyopathy (HCM), due to its genetic aetiology with dominant autosomal transmission and complete or incomplete penetrance, became a central subject in cardiology although it presents a highly variable phenotypic, clinical and prognostic heterogeneity [1]. Around 65% of HCM patients have relatives with either genotype-positive/phenotype-positive (g+p+) or genotype-positive/phenotype-negative (g+p-) status and more than 1400 mutations carried by more than 13 genes were identified [2]. The two most common mutations include myosine binding protein C3 (MYBPC3) and myosine heavy chain (MYH7) with an overall 70% prevalence whereas troponin T2 (TNNT2) mutations represent about 7% prevalence and other mutations are quite rare [2,3].

Despite conserved ejection fraction (EF), systolic phase undergoes functional impairment in HCM patients: magnetic resonance (RMN) and echocardiography are the most used diagnostic tools and in particular speckle tracking echocardiography in either 2- or 3-dimensions (2DSTE, 3DSTE) might be of great value [4]. Systolic dysfunction is evaluated by calculating strains, twist and rotations whereas diastolic abnormalities are more difficult to be seen [5]. Longitudinal, radial and circumferential strains were attenuated and useful to index HCM patients as compared to healthy individuals [6,7]. On the other hand, it was shown that some rotational parameters and trajectory attributes [5,8] are delayed during diastole in comparison to healthy individuals, thus suggesting that also LV diastolic function is compromised in HCM patients. Therefore, evaluating LV revolution during heart cycle might unveil new patterns in HCM pathophysiology. More importantly, these investigations could be applied to g+p- individuals in order to assess if their “static” global shape or the shape of their LV morphological trajectories are discriminant as compared to healthy individuals. Our approach allows answering important physiological questions: a) given the intimate relationship “form and function”, which of these undergoes first the most serious modifications in affected individuals? b) which of these is the most resistant to alterations caused by HCM? c) is epicardium or endocardium in HCM patients the layer most setting apart from healthy individuals? d) is (and how much if any) the natural epi-endocardial synchronism altered in HCM patients? e) is there any means to assess quantitatively direct diastolic indicators in HCM? f) is there any means to index genotype from phenotype in HCM? Question e) and f) were faced recently using echocardiographic studies. Ho et al [9] found some significant parameters in g+p- individuals using Doppler Tissue Imaging thus suggesting that diastolic abnormalities are an early subclinical manifestation of HCM, which precedes development of LV hypertrophy. More recently, Gruner et al [10] used a variety of clinical and basic echocardiographic indicators to predict genotype in a wide sample of subjects. Several authors provided a huge amount of studies (whose complete review is not the scope of the present paper) illustrating functional LV impairments in HCM patients. However, none of these used modern shape deformation analysis to evaluate LV shape changes in HCM and g+p- individuals via speckle tracking. This approach could help diagnosis and prevention by looking for new preclinical indicators also during diastole.

Recently, Piras et al. [8] proposed a new method for the evaluation of LV motion using Procrustes Motion Analysis and a modified Geometric Morphometrics toolkit in order to study not only the shape of LV but also the shape, size and orientation of its trajectory in time. Their approach used the concept of homologous times to identify comparable time frames at which the LV shape is predicted for reconstructing its motion trajectory. A proper morphospace is then built to correctly compare different trajectories by means of the “linear shift” strategy [8]. In the present investigation we aimed at comparing LV trajectories of HCM individuals to assess the shape change pathophysiology underlying HCM as well as the attributes of its motion trajectories when contrasted with healthy subjects. We evaluated the performance of both static shape analyses and trajectory analysis by comparing them with classical global ecocardiographic indicators. Moreover, we preliminary included 3 g+p- subjects as HCM family members of patients included in our overall sample to explore the promise of the new approach proposed here.

## Methods

### Subjects and Ethic statements

The study was conducted after the approval of the ‘‘Dipartimento di Scienze Cardiovascolari, Respiratorie, Nefrologiche, Anestesiologiche e Geriatriche, Sapienza-Università di Roma” and in accordance with the ethical guidelines of the Declaration of Helsinki. Written informed consent was obtained from each subject. From April 2012 to April 2014, a total of 75 subjects were studied. For 48 healthy subjects we assessed, based on an accurate cardiological visit, the absence of any type of known heart disease. There were 24 non-obstructive HCM patients. Table 1 shows descriptive statistics of the sample under study. Moreover, there were 3 g+p- (1 with MYBPC3, 1 with MYH7 and 1 with TNNT2) family members of the HCM patients included as a preliminary approach.

**Table 1:** Descriptive statistics ± standard deviation for the populations under study.

Descriptive parameters	Control = 48	HCM = 24
Age	39±8.64	47±12.39
Ejection Fraction	59.1±0.05	54.8±0.08
Inter-Ventricular Septum	8.48±1.41	18.16±3.84
Males/Females	32/16	14/10
Beat rate	77±13.16	75±13.00
**Genetic Mutations for HCM**		
MYBPC3	-	7
MYH7	-	4
TNNT2	-	3
Mutation not known	-	6
Not investigated	-	4

### 3D data acquisition

**Fig 1:**
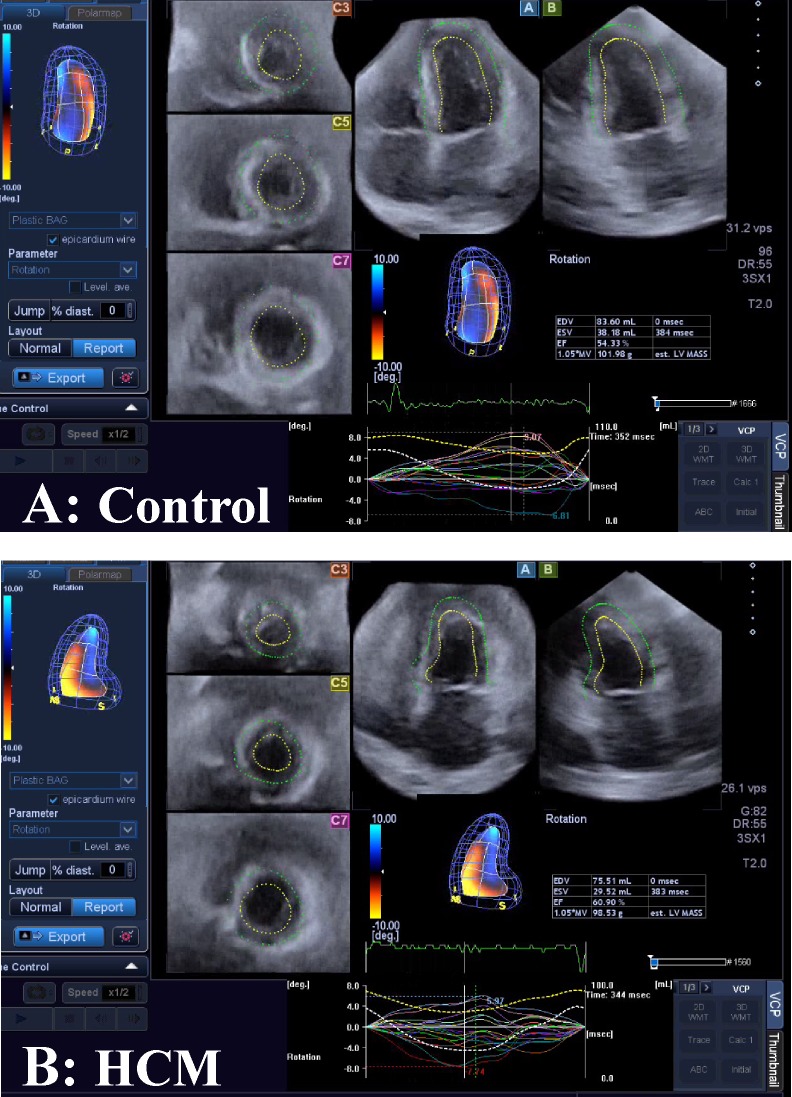
The Speckle Tracking methodology. (A) Healthy control subject. (B) Individual affected by Hypertrophic Cardiomyopathy.

Our methods derive basically from Piras et al [8]. They introduced a new strategy for studying motion applied to LV geometry moving in real time. Initially, the acquisition of LV geometry is achieved by means of 3DSTE, a technique that became a gold standard in cardiological diagnosis as it allows the real time evaluation of LV motion and the measurements of several diagnostic parameters such as twist, torsion, rotation among others. We collected LV shape data by means of PST–25SX Artida device, Toshiba Medical Systems Corp., Tokyo, Japan. The LV geometry is reconstructed starting from a set of 6 homologous landmarks, manually detected by the operator for all subjects under study. The same operator (AE) was involved in LV geometry reconstruction (Fig 1).

The manual detection for a set of landmarks is important as it allows recording the spatial coordinates in comparable anatomical structures of different subjects following a homology principle [11]. Fully automated approaches suffer from error of pattern identification depending on specific algorithms used for reconstruction [12–15]. The final dataset of any subject is a time-sequence of shapes, each constituted by 1297 landmarks (assumed to be homologous) for either the epicardial and endocardial surfaces, positioned along 36 horizontal circles, each composed of 36 landmarks, plus the apex. It was possible to obtain the landmark cloud (upon which the standard rotational, torsional and strain parameters are computed and outputted by each Artida device) by an unlocked version of the software equipping our PST–25SX Artida device, thanks to a special opportunity provided in the context of an official research and development agreement between the Dipartimento di Scienze Cardiovascolari, Respiratorie, Nefrologiche Anestesiologiche e Geriatriche, ‘‘Sapienza” Università di Roma and Toshiba Medical System Europe, Zoetermeer, The Netherland.

### Procrustes Motion Analysis

**Fig 2:**
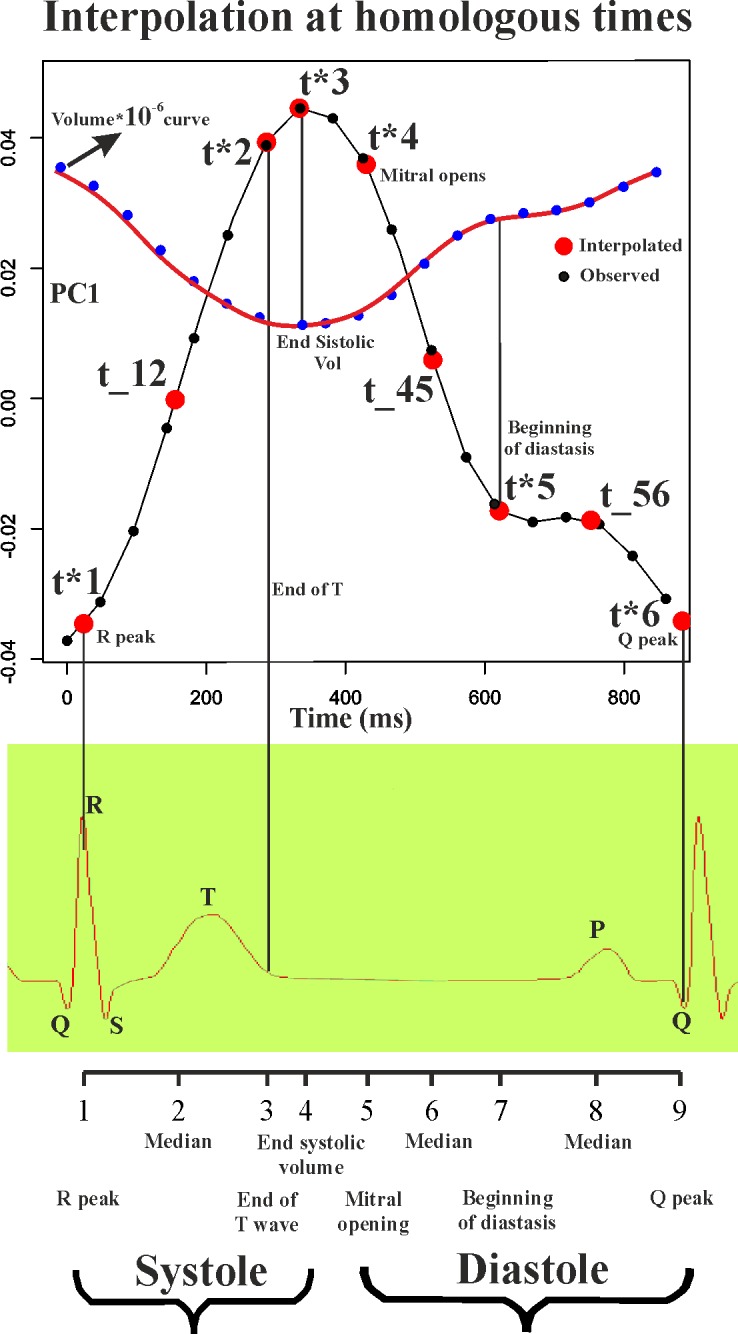
Interpolation of shape variables (here the sole PC1 is shown) at electrophysiological homologous times. Note that homologous times are not equally spaced during cardiac revolution.

The mean temporal resolution of our data is about 40 ms with a standard deviation of 6.19 ms. It means that, on average, about 20 LV shapes changing in time are recorded within a single beat. However, any subject is represented by a different number of LV shapes (min: 12; max: 33) captured within a single heart cycle depending on individual heart beat rate. In order to properly define a set of motion trajectories constituted by the same number of shapes, we therefore adopted the same strategy described in Piras et al. [8]. Our approach takes advantage of the manual identification of time frames (expressed in ms) at which homologous electromechanical events occur within any subject under study. This temporal homology is absolutely critical as is anatomical homology in morphological studies [16]. In fact, comparing the attributes of a set of trajectories (i.e. their shape, size and orientation) implies that the morphology changing in time must be captured at physiologically homologous times. Heart revolution offers the unique opportunity for recognizing events that are specifically homologous under electrical and mechanical points of view. Apart end systolic volume and few others, there were no previously constantly considered homologous electromechanical times in clinically oriented investigations before Piras et al. [8] whom used a complete set of homologous times in order to study the shape of the morphological revolution itself. We then choose from visual inspection of the electrocardiogram and echocardiographic videos associated to each 3DSTE registration, 3 electrical events (onset of R, end of T, and Q minimal waves) and 3 mechanical ones (end systolic volume, mitral-valve opening, end of rapid filling/beginning of diastasis) to obtain a sequence of electrophysiologically homologous times for each subject i: {t*1, t*2, t*3, t*4, t*5, t*6}i, with t*1 = R peak; t*2 = end of T wave; t*3 = end systolic volume; t*4 = mitral-valve opening; t*5 = end of rapid filling/beginning of diastasis; t*6 = Q min. We specify here that the end systolic volume time is assumed to be coincident (thus without error) with that provided by the Artida device. Thus, LV shape at this time coincides with that estimated by the machine at that time. Given a mean temporal resolution of 40 ms, it implies that the error is ±20 ms. This approximation is identically accepted by every experimental study published till now worldwide, using a 3DSTE. Additionally, for a better interpolation of shapes over time, we added 3 median points to the above-mentioned sequence: t_hk_ = median point between t*_h_ and t*_k_. Thus, the final sequence of homologous times for subject i^th^ comprises 9 times: ht_i_ = {t*1, t1_2, t*2, t*3, t*4, t4_5, t*5, t5_6, t*6}. Fig 2 summarizes the interpolation procedure described above.

Once assessed the 9 time frames for any subject, we proceeded as follows to predict LV shapes at these times for each individual. We denote here that all analyses described below were performed on epicardial and endocardial shapes separately. This is crucial because considering the two layers together fatally leads to a serious circular bias: the layers include the myocardial thickness that is just the parameter upon which the HCM pathology is defined. Analyzing them separately eliminates this circularity and will lead to differential identification of functional impairments affecting LV inner and outer strata. First, we performed *separate* Generalized Procrustes Analyses (GPA) for every subject in the dataset and a corresponding Principal Component Analysis (PCA). We carefully note that GPAs performed here or in the linear shift (see below) are performed in both *size and shape space* [17], i.e. without scaling all subject to the same size, and in the *shape space* thus removing size as commonly done in many Geometric Morphometrics applications. The two approaches are compared and contrasted: the first reason for this stems from the fact that contraction, thus reduction in size, represents an important parameter of heart function. Size represents a genuine mechanical deformation even when it does not affect shape. Moreover, in the case of data subjected to a linear shift (see below), together with centering in shape, the size also is centered on the Grand Mean, thus maintaining intra-individual size excursion but filtering out inter-individual size differences. We then first perform separate (per-individual) GPAs+PCAs on the size and shape space (size must be maintained at this stage: it can optionally be maintained or partialled out in the successive steps) and we predicted, for any subject separately, all PC scores explaining up to 100% of total variance in correspondence of individual specific homologous time frames. We performed this prediction by using a cubic spline interpolation (using spline() function in R package ‘‘stats”) on the relationships between time (in ms, as independent) and values of the PC scores (as dependents). In some cases the homologous times are very close to the times at which the machine acquires the data. Although the 3D frame rate has a lower resolution as compared to a 2D acquisition rate (~40 ms vs. ~20 ms, respectively), just by chance some of our homologous times could coincide with the actual acquisition by the machine and those who may not be coincident might have a 20 ms maximum error, which in any case is a very tiny difference as compared to the global systolic duration (~450 ms). As for the end systolic volume, we assume (as stated before) that the machine acquisition represents the true value. Successively, using all PC scores, we reconstructed the original LV shapes by using the subject specific eigenvalues and eigenvectors computed during individual specific PCAs. At this stage we then have, for any subject, a set of 9 epicardial and 9 endocardial shapes (each constituted by 1297 landmarks in 3 dimensions) representing LV morphology captured at homologous electromechanical events. We applied the new strategy of linear shift, described in Piras et al. [8] to these data. The linear shift is essential in order to filter out initial inter-individual variability and leaving just the motion path differences. The shifted data can be then subjected to a standard GPA+PCA in the size and shape space or in the shape space. We used the values of the first 3 PC scores coming from this last PCA as homologous landmarks representing 3-dimensional shapes of LV trajectories. Then, these shapes are constituted by 9 landmarks in 3 dimensions representing the LV trajectory in time. This step represents a second-order level of abstraction as these shapes are no longer LV shapes but the shapes of LV trajectories themselves. We stress here that trajectory attributes are not related to specific homologous times as they characterize the entire LV revolution evaluated during the whole heart cycle. When testing orientation we choose the angles between PC1-PC2 and PC1-PC3 using the PC scores of shapes predicted at the first and fourth times that correspond to R-peak and end systolic volume, respectively. Testing the shapes of the trajectories, we performed a GPA followed by a PCA in order to evaluate trajectories shape and size as done in Adams and Collyer [18], Collyer and Adams [19], Collyer et al [20] and Piras et al. [8]. Median points cited above were excluded during Procrustes Distance minimization process and were passively appended to transformations (translation, scaling and rotation) applied to PC values estimated at any t* electromechanical homologous time. This strategy allows to compare relatively complete shapes of trajectories without adding noise due to non perfectly homologous physiologically-based event estimation. For size of trajectories we computed the sum of phenotypic distances between any point interpolated for each trajectory.

### Static shape analysis

For any homologous time we performed GPAs+PCAs in order to explore morphological differences between healthy subjects and HCM patients. We evaluated also, at any homologous times, the shape differences for each individual from his proper shape at R peak, i.e. in telediastole. These analyses were performed using respectively pure shapes in the size and shape space, shapes transported in the size and shape space and shapes transported in the shape space. We also performed corresponding UPGMA cluster analyses to explore the relationships between healthy, HCM and g+p- individuals that evolve differently within the heart revolution. UPGMAs were performed on euclidean distances among the 3 groups. Albeit these analyses are properly static analyses of shape change, their meaning is profoundly dynamic, as they are performed at electrophysiologically homologous times and, as a consequence, they should be evaluated as whole time sequence.

### Morphological integration

Endocardium and epicardium inevitably move together and they covary during cardiac revolution. However, this covariation could (or could not) undergo significant alteration when comparing healthy to pathological individuals. Moreover, as the landmarks of the speckle tracking technology are virtually anchored to epicardial and endocardial tissues moving in time, they can be used in order to assess which layer sets apart the most from healthy conditions during LV cycle. A potentially important consequence of applying this interpretation could be that of regionalization of myocardial disarray on one hand and that of the lever arm length (and curvature) in the functional couple epicardium-endocardium [21,22] on the other. To test these explicit hypotheses we use a conventional metrics used in Geometric Morphometrics: the RV coefficient. RV coefficient is the multivariate version of the coefficient of correlation [23] and varies from 0 to 1. As first, we assessed within any patient the RV coefficient between endocardium and epicardium using the 9 epicardial and endocardial shapes at the corresponding homologous times. Then we used a permutated ANOVA to test for difference in RV between healthy subjects and HCM patients. This was aimed at testing if epicardium and endocardium are somewhat “desynchronized” in HCM patients. Another important inference was done by comparing, separately, the epicardial and endocardial end systolic shapes of HCM patients versus healthy subjects. This was done on data transported (that we recall represent the deformation, not shapes *per se*) in the shape space thus filtering out size. This choice is justified upon the following reasoning: in investigating the differential fate of epicardium and endocardium during contraction and the consequences of HCM disease on their size and shape changes, it is useful to know how much the global change is driven by homothetic contraction and how much pertains to pure shape change. This is important because the muscular disarray in HCM patients implies an inefficient contraction. This inefficiency could just decrease contraction (in homothetic sense) or also entail a smaller change in terms of pure shape. As size is essential during LV revolution, its removal is important for identifying where the effect of pathology takes place, i.e on a reduced volumic contraction or also on pure shape.

Being shape multivariate, we build a 2 way permutated MANOVA design (employing the first 10 PC scores values of data transported in the shape space at end systolic time) using control sample and HCM patients as primary factor and endocardium-epicardium as the secondary one. We then evaluated their interaction. In order to compare the design made on shape variables with the behaviour of size, we performed the same analysis using the differences in size from diastole and systole by means of an univariate 2 way ANOVA. We speculate that knowing where interaction is significant could help in identifying (together with other observations) on which layer (epicardium or endocardium) and on which attributes (pure shape or size) the anomalies due to HCM disarray operate.

### Linear models

We used permutated MANOVA and ANOVA in order to test differences between the control sample and HCM subjects for the various indicators we described above. Successively, we used logistic regression, with pathological status coded as binary dependent, in order to evaluate the performance of a different set of independent variables in discriminating healthy subjects from HCM patients. Different models are then built depending on the different nature of predictors. For any homologous time (t = 9) we evaluated separately all univariate descriptive parameters outputted from 3DSTE (global strains, rotations and displacements) and the multiple set of first 5 PC scores coming from static shape analyses. We chose only 3DSTE global parameters because our morphometric analyses are performed on entire endocardial and epicardial shapes. It is for further studies the subcomparison according to 16-segments ASE localization [24].

For static shape analyses (epicardium and endocardium), we evaluated the pure shapes in the size and shape space, the shapes transported in the size and shape space, the shapes transported in the shapes space and, for any of these datasets, the shape differences at any homologous time from the shape at the R peak, i.e. in the same manner the 3DSTE parameters are computed. Eventually, we also used the trajectory attributes: the multiple set of PC scores of trajectory shape analysis and the univariate angles defined by PC1-PC2 and PC1-PC3 proper of each subject-specific trajectory. We also used RV coefficient computed for any individual between epicardium and endocardium along the entire LV revolution. These last analyses cannot be related to single homologous times as they come from the evaluation of trajectory attributes, thus during the entire temporal LV revolution. As these models are not nested and can have different number of predictors, the R-square cannot be considered as a metric for model performance comparison. We choose, for critical evaluation of our logistic regression models, the Akaike Information Criterion [25]. It represents the amount of information lost during model construction and it accounts for the number of predictors. The smaller AIC indicates the preferred model. Using predicted values probabilities coming from the above mentioned logistic regressions, we built a corresponding number of ROC curves and derived the “area under the curve” (AUC). The AUC is often used as a measure of discrimination performance of different indicators or treatments. We contrasted the best 3DSTE global indicators in systole and diastole with our new morphometric indicators. Additionally, we contrasted AUCs of epicardium and endocardium shapes transported in both size and shape space and shape space at 1^th^, 4^th^ and 7^th^ homologous times thus covering the entire LV revolution. We used De Long et al. [26] method in order to assess if 2 ROC curves are statistically different.

### The relationship between Geometric Morphometrics indicators and 3DSTE parameters

In order to explore the meaning of the above mentioned indicators when compared to 3DSTE parameters, we calculated the coefficient of correlation between 3DSTE global variables evaluated at end systole with all morphometric indicators coming from the analyses described above. As there is no specific time points whereby trajectory attributes are calculated, as previously stated, PC scores coming from static analysis, linear shifts and shape differences from the R peak were instead correlated using PC values corresponding to end systolic time.

### The preliminary inclusion of 3 g+p- individuals

The existence of g+p- individuals represents a challenge for HCM diagnosis and prevention. The asymptomatic state of these subjects might force to genetic analysis solely on the basis of HCM presence in their family history. By definition, their “phenotypic negativity” usually makes classic echocardiography of scarce utility in their clinical identification. The logic behind the inclusions of these 3 g+p- individuals rests on a preliminary assessment of the promise of modern shape analyses we present here. In fact, coupling static shape analysis with the notion of homologous times, will allow exploring their placement in ordination methods relatively to healthy individuals and HCM patients in different phases of cardiac revolution, i.e. from systole to diastole. An echocardiography-based method potentially able to predict the genotype state in individuals “phenotipically negative” could be of high impact for their identification before and independently from genetic analyses. Being the g+p- sample so small we just run an explorative cluster analysis without testing explicit hypotheses as done for HCM patients. These analyses were done on pure epicardial and endocardial shapes in the size and shape space, on shapes transported in the size and shape space and shapes transported in the shape space.

## Results

### Deformation analysis

**Fig 3:**
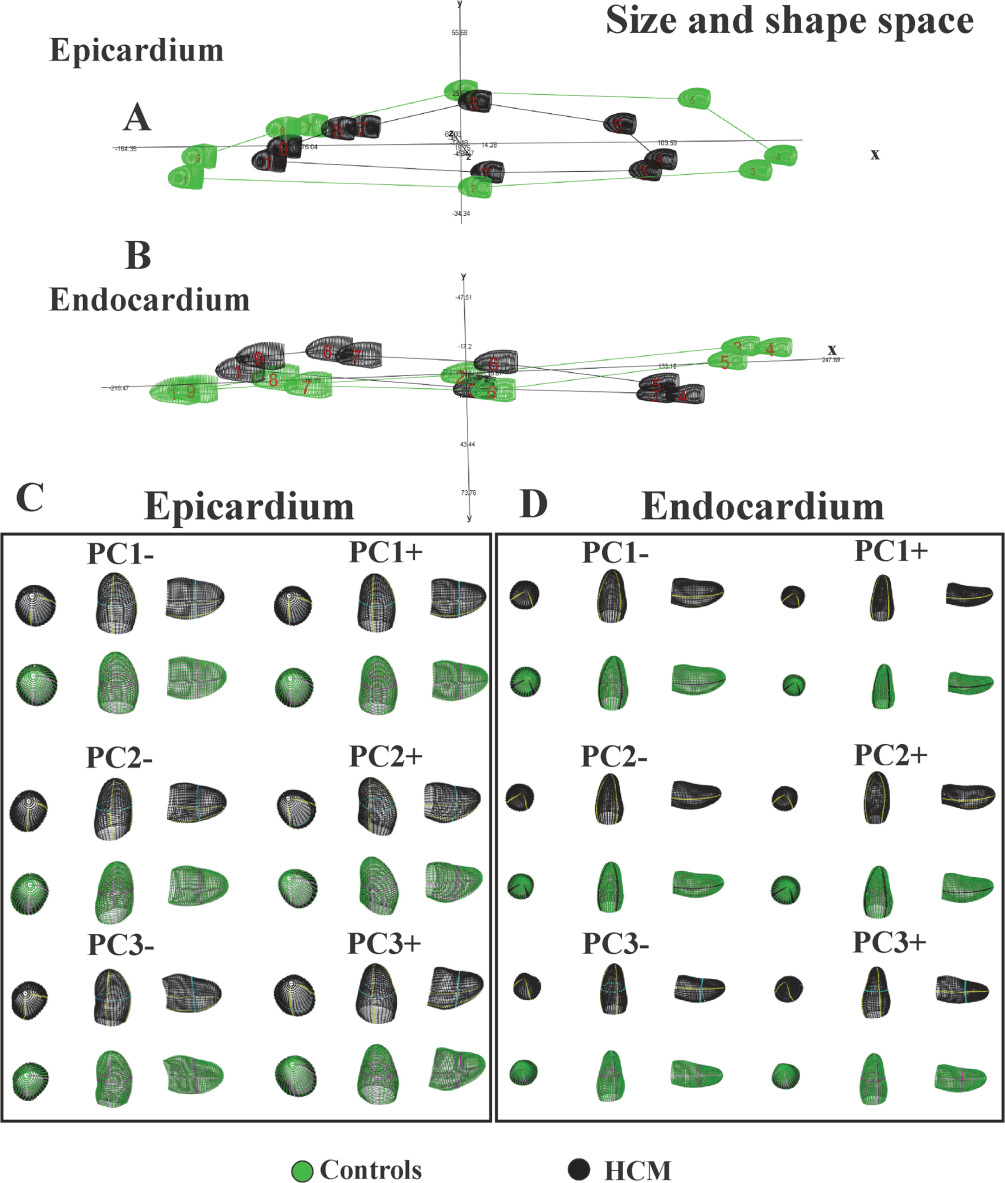
The shape of trajectories and their morphological meaning in the size and shape space. (A) Epicardial trajectory. (B) Endocardial trajectory. (C) Shape changes explained by the first three PC scores of epicardium. (D) Shape changes explained by the first three PC scores of endocardium. Animated GIF of these figures are in the Supporting Information as well as the corresponding figures of data transported on the shape space.

Results of GPA+PCA performed on data shifted in the size and shape space are presented in Fig 3 that shows both epicardial and endocardial trajectories.

We recall that these shape changes express solely the deformations not influenced from shapes to which they apply. Animated version of these shape changes can be found in S1–S20 Figs. In this Supporting Information we also show the same results in the shape space thus not considering size. We urge the reader to appreciate both the shape of the trajectories and the shape changes explained by PCs illustrated in the above mentioned animations. As we performed analyses of Fig 3 in the size and shape space, it is evident that the PC1 explains LV contraction while PC2 and PC3 are genuine shape changes not influenced by size. Individual deformations along single PCs are useful in separating the contraction from pure shape changes. Profound differences are visible passing from epicardium to endocardium. While both PC1s illustrates a pure volume contraction, the other PCs tell us very different stories for the two layers. For endocardium we see a shearing along PC2 and a bending-rotation along PC3. Healthy and HCM individuals differ, as it can be seen on the 3D plot of epicardial trajectory, mainly for the magnitude of their variations along PCs. On the opposite, for endocardium, PC2 and PC3 point in opposite directions, passing from systole to diastole, in the 2 groups. Endocardial PC2 shows a shape lengthening that is inverted in HCM patients. The same signal is evident along PC3 that explains a light basal expansion that tends to disappear in systole. These dramatic differences between control and HCM individuals are also evident in the trajectory 3D plot that clearly shows healthy and pathological trajectories that cross each other.

### Trajectory attributes analysis

**Fig 4:**
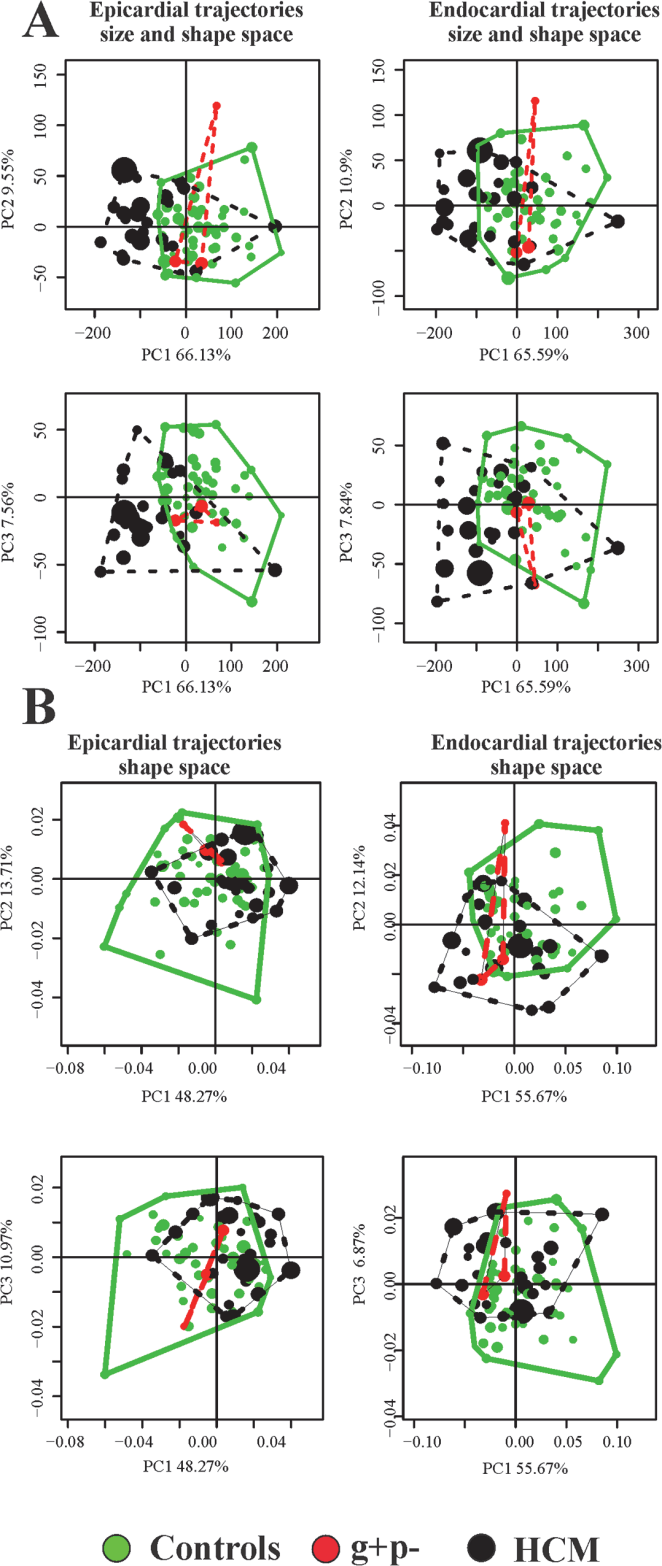
PCA analyses performed on the shape of the trajectories themselves. **PC1-PC2 and PC1-PC3 scatterplots are shown.** (A) Size and shape space. (B) Shape space.

**Fig 5:**
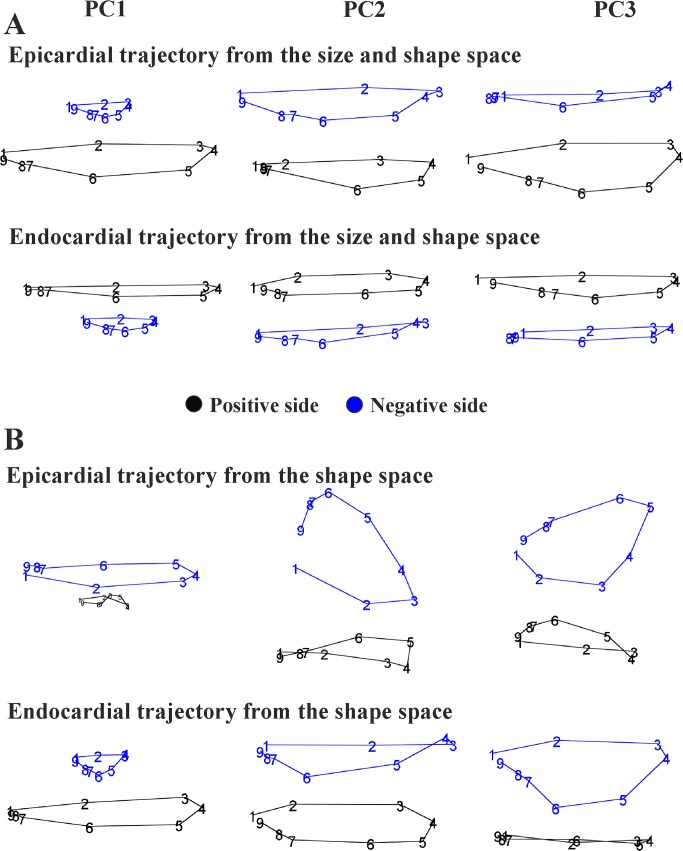
The shape of trajectories, constituted by the nine values of first three PC scores of LV shape interpolated at the nine homologous times, explained by the first three PC scores of the LV trajectory shape analyses. (A) LV trajectories identified in the size and shape space. (B) LV trajectories identified in shape space. We stress here that these PC scores are referred to the shape of the trajectories themselves not directly to the shape of LV.

Values of first 3 PC scores coming from the linear shift at any homologous times were treated as homologous landmarks and subjected to GPA+PCA. Fig 4 and Fig 5 show results of these analyses and the trajectories shapes explained by the first 3 PC scores of both size and shape space and shape space.

For both the size and shape space and shape space, the PC1 represents the size of the trajectories (correlation with trajectory size: R square = 0.99; p value = 0.001 for both epicardium and endocardium) with the majority of the variance explained, while the other PCs are associated to pure shape deformations not related to size. Table 2 shows results relative to shape, size and orientation of trajectories when analyzed under permutated ANOVA and MANOVA models using control/HCM as factor.

**Table 2:** Trajectories attributes ANOVA and MANOVA results for differences between the control sample and HCM individuals.

Epicardium size and shape space	*p*-value
Size	**0.001**
Shape (first five PCs)	**0.001**
PC1-PC2 angle	**0.015**
PC1-PC3 angle	**0.013**
**Endocardium size and shape space**	
Size	**0.001**
Shape (first five PCs)	**0.001**
PC1-PC2 angle	**0.001**
PC1-PC3 angle	**0.001**
**Epicardium shape space**	*p*-value
Size	**0.002**
Shape (first five PCs)	**0.006**
PC1-PC2 angle	**0.02**
PC1-PC3 angle	**0.01**
**Endocardium shape space**	
Size	0.07
Shape (first five PCs)	**0.014**
PC1-PC2 angle	**0.001**
PC1-PC3 angle	**0.001**

In bold significant results.

**Fig 6:**
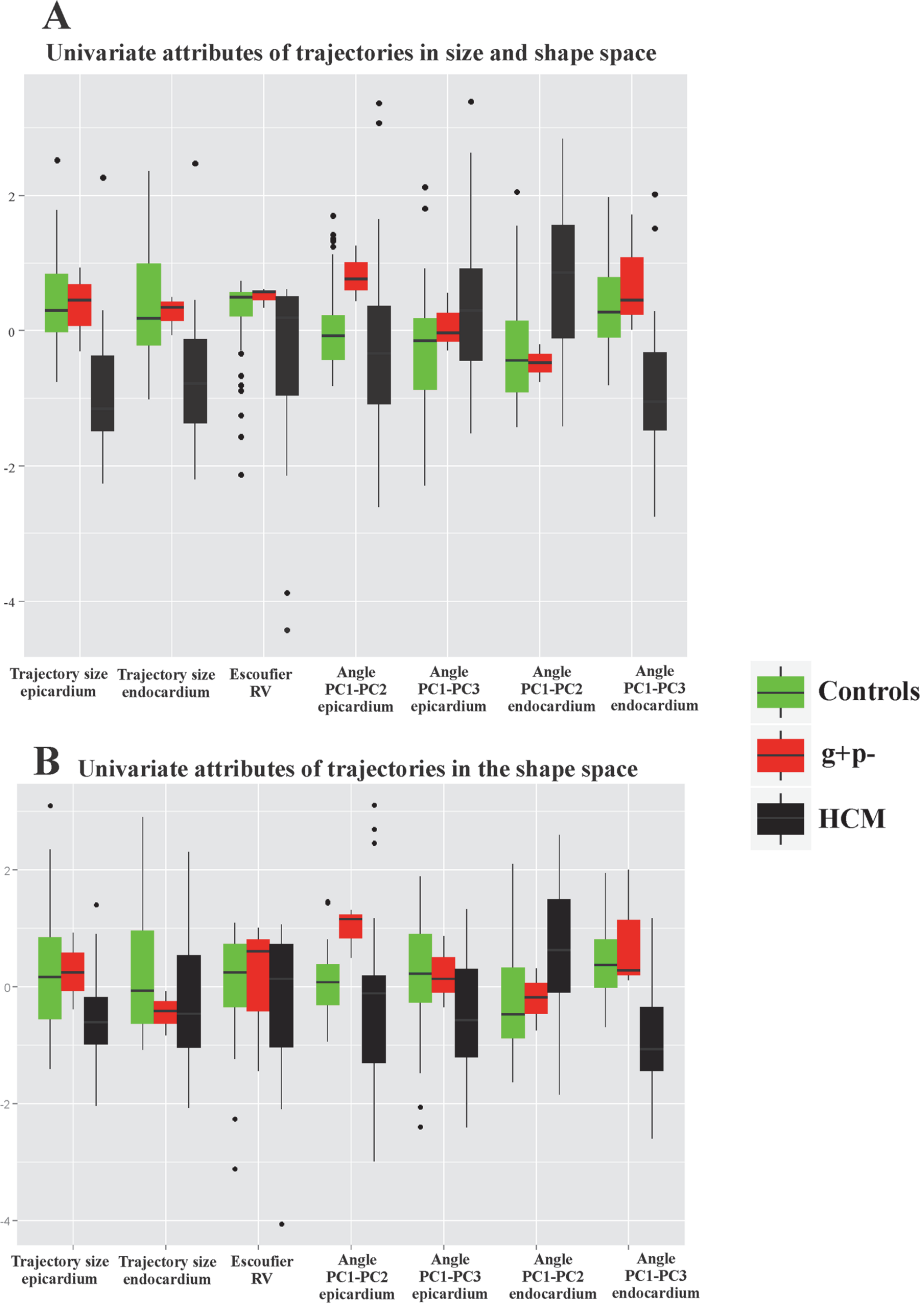
Box plots of the univariate attributes of trajectories. (A) Size and shape space. (B) Shape space.

All parameters resulted highly significant. The differences in multivariate shape of trajectories were significant even eliminating the PC1 that represents trajectory size. HCM patients are shifted along PC1 toward values corresponding to smaller trajectories (Fig 4). However, among the first 5 PC scores, looking at differences among single PCs, we found no significant differences among the PC2, while the PC3 shows significant differences between control and HCM individuals. As it can be seen in Fig 5, the sides of PCs at which HCM individuals take place in Fig 4 correspond to smaller and less rounded trajectories in comparison to shapes proper of control sample positions. Fig 6 presents in box plots the distributions of univariate attributes of morphological trajectories in the different groups for both size and shape space and shape space.

For all these parameters, except for PC1-PC2 angle of epicardial trajectory, the comparison between control and HCM individuals is statistically significant under permutated ANOVA. An interesting result regards the size of endocardial trajectory shape that is significantly larger than that of epicardium (*p*-value: 0.001). This holds also for data transported in the shape space thus not considering size. It means that endocardium changes more than epicardium, during its revolution, in terms of both size and pure shape.

### Linear models

Results of logistic regressions (using control/HCM as a binary response), are shown in Table 3 and Table 4.

**Table 3:** Logistic regression AIC and *p*-values for the 9 homologous times for 3DSTE parameters.

Toshiba Artida 3DSTE parameters	R peak	End of T wave	Median	End systolic volume	Mitral opening	Median	Beginning of diastasis	Median	Q peak
Radial Strain_global	95.64	93.36	88.51	89.97	91.24	93.89	95.41	93.31	92.28
Circumferential Strain_global	95.65	94.83	80.1	78.43	83.76	91.42	94.84	95.24	95.59
Longitudinal Strain_global	95.22	86.47	65.05	65.58	76.98	93.42	95.41	95.66	95.6
Rotation_global	95.66	90.23	90.35	92.16	94.31	93.27	95.14	93.49	92.6
Twist_global	94.5	95.09	95.57	95.33	94.05	93.78	94.29	93.25	93.55
Torsion_Regional_global	95.43	94.78	92.59	93.06	95.55	94.4	92.62	90.39	91.36
Torsion_Basal_global	94.23	95.4	95.56	95.33	94.21	94.37	95.16	94.59	94.69
Strain_3D_global	95.61	88.64	82.1	84.45	85.73	89.64	95.39	95.43	94.42
Radial Displacement_global	94.11	95.24	89.34	89.59	92.4	94.11	95.51	95.65	95.65
Longitudinal Disp_global	90.85	64.77	60.27	64.61	74.22	94.01	95.44	93.67	94.26
X3DDisp_global	95.49	88.49	73.52	76.01	83.87	94.86	95.64	95.61	95.64
AreaTracking_global	95.57	92.36	74.71	72.84	80.72	91.78	95.45	95.48	95.66
**Corresponding *p*-values**									
Radial Strain_global	8.86E-01	1.44E-01	**1.46E-02**	**2.57E-02**	**4.60E-02**	1.94E-01	6.17E-01	1.33E-01	7.48E-02
Circumferential Strain_global	9.54E-01	3.67E-01	**1.50E-03**	**1.34E-03**	**3.34E-03**	5.18E-02	3.67E-01	5.19E-01	7.88E-01
Longitudinal Strain_global	5.08E-01	**9.04E-03**	**6.45E-05**	**6.25E-05**	**3.92E-04**	1.47E-01	6.22E-01	9.81E-01	8.13E-01
Rotation_global	9.58E-01	**2.60E-02**	**2.97E-02**	**7.17E-02**	2.52E-01	1.34E-01	4.73E-01	1.50E-01	9.51E-02
Twist_global	3.08E-01	4.58E-01	7.61E-01	5.68E-01	2.12E-01	1.77E-01	2.47E-01	1.33E-01	1.68E-01
Torsion_Regional_global	6.32E-01	3.53E-01	9.32E-02	1.20E-01	7.47E-01	2.72E-01	9.13E-02	**3.10E-02**	5.59E-02
Torsion_Basal_global	2.52E-01	6.11E-01	7.57E-01	5.68E-01	2.36E-01	2.62E-01	4.82E-01	3.05E-01	3.33E-01
Strain_3D_global	8.34E-01	**1.63E-02**	**1.83E-03**	**3.38E-03**	**5.06E-03**	**2.34E-02**	6.05E-01	6.34E-01	2.70E-01
Radial Displacement_global	2.19E-01	5.20E-01	**2.00E-02**	**2.35E-02**	**8.52E-02**	2.25E-01	6.98E-01	9.52E-01	9.09E-01
Longitudinal Disp_global	5.68E-02	**3.38E-05**	**2.22E-05**	**2.75E-05**	**1.04E-04**	2.08E-01	6.38E-01	1.67E-01	2.45E-01
X3DDisp_global	6.78E-01	**1.30E-02**	**1.46E-04**	**2.79E-04**	**2.16E-03**	3.76E-01	9.08E-01	8.35E-01	8.97E-01
AreaTracking_global	7.71E-01	7.77E-02	**4.93E-04**	**4.55E-04**	**1.42E-03**	5.98E-02	6.47E-01	6.69E-01	9.62E-01

In bold significant results.

**Table 4:** Logistic regression AIC and *p*-values for the 9 homologous times for LV shape static parameters.

	R peak	End of T wave	Median	End systolic volume	Mitral opening	Median	Beginning of diastasis	Median	Q peak
**LV shapes in size and shape space**									
Epicardial shape	69.77	72.28	81.21	81.82	76.25	66.20	69.76	69.29	68.28
Endocardial shape	70.40	71.10	81.53	82.79	80.26	73.72	74.96	74.86	70.72
Epicardial shape differences from R peak	93.66	86.16	63.28	62.96	73.53	84.64	92.54	93.11	95.65
Endocardial shape differences from R peak	93.66	91.60	74.18	75.10	81.10	88.08	95.01	94.97	95.58
**Corresponding *p*-values**									
Epicardial shape	**2.51E-06**	**7.90E-06**	**4.30E-04**	**5.61E-04**	**4.75E-05**	**4.86E-07**	**2.49E-06**	**2.02E-06**	**1.27E-06**
Endocardial shape	**3.34E-06**	**4.61E-06**	**4.95E-04**	**8.57E-04**	**2.84E-04**	**1.52E-05**	**2.66E-05**	**2.53E-05**	**3.87E-06**
Epicardial shape differences from R peak	NA	**2.06E-03**	**1.27E-08**	**1.08E-08**	**2.55E-06**	**9.05E-04**	7.75E-02	1.10E-01	9.49E-01
Endocardial shape differences from R peak	NA	**4.39E-02**	**3.59E-06**	**5.79E-06**	**1.36E-04**	**5.92E-03**	4.21E-01	4.08E-01	7.77E-01
**LV shape transported in the size and shape space**									
Epicardial transported data	60.29	56.13	45.04	50.90	72.21	60.45	60.76	69.62	61.89
Endocardial transported data	34.17	73.35	41.16	44.90	42.99	81.51	72.12	70.66	56.93
Epicardial transported data differences from R peak	93.66	83.88	57.61	57.57	66.67	75.69	88.00	89.38	95.56
Endocardial transported data differences from R peak	93.66	90.29	71.07	72.08	78.21	84.92	94.77	94.85	95.60
**Corresponding *p*-values**									
Epicardial transported data	**3.11E-08**	**4.44E-09**	**2.34E-11**	**3.76E-10**	**7.64E-06**	**3.35E-08**	**3.88E-08**	**2.34E-06**	**6.57E-08**
Endocardial transported data	**1.31E-13**	**1.28E-05**	**3.69E-12**	**2.20E-11**	**8.83E-12**	**4.91E-04**	**7.34E-06**	**3.77E-06**	**6.45E-09**
Epicardial transported data differences from R peak	NA	**6.00E-04**	**6.90E-10**	**6.78E-10**	**7.28E-08**	**7.89E-06**	**5.67E-03**	**1.22E-02**	7.50E-01
Endocardial transported data differences from R peak	NA	**2.05E-02**	**7.10E-07**	**1.20E-06**	**2.95E-05**	**1.05E-03**	3.45E-01	3.69E-01	8.07E-01
**LV shape transported in the shape space**									
Epicardial transported data	79.77	51.98	44.92	62.18	79.80	61.47	56.31	64.70	68.34
Endocardial transported data	43.58	74.89	45.44	44.72	41.95	83.15	75.46	72.67	61.34
Epicardial transported data differences from R peak	93.66	95.00	87.49	86.50	86.87	85.86	90.43	92.00	95.38
Endocardial transported data differences from R peak	93.66	94.70	91.34	90.80	89.28	88.19	93.26	93.26	95.61
**Corresponding *p*-values**									
Epicardial transported data	**2.29E-04**	**6.28E-10**	**2.22E-11**	**7.50E-08**	**2.31E-04**	**5.38E-08**	**4.83E-09**	**2.42E-07**	**1.30E-06**
Endocardial transported data	**1.17E-11**	**2.58E-05**	**2.84E-11**	**2.01E-11**	**5.40E-12**	**1.00E-03**	**3.33E-05**	**9.42E-06**	**5.09E-08**
Epicardial transported data differences from R peak	NA	**4.16E-01**	**4.26E-03**	**2.48E-03**	**3.02E-03**	**1.75E-03**	**2.22E-02**	5.58E-02	6.01E-01
Endocardial transported data differences from R peak	NA	**3.27E-01**	**3.77E-02**	**2.76E-02**	**1.15E-02**	**6.27E-03**	1.21E-01	1.21E-01	8.35E-01

In bold significant results.

**Fig 7:**
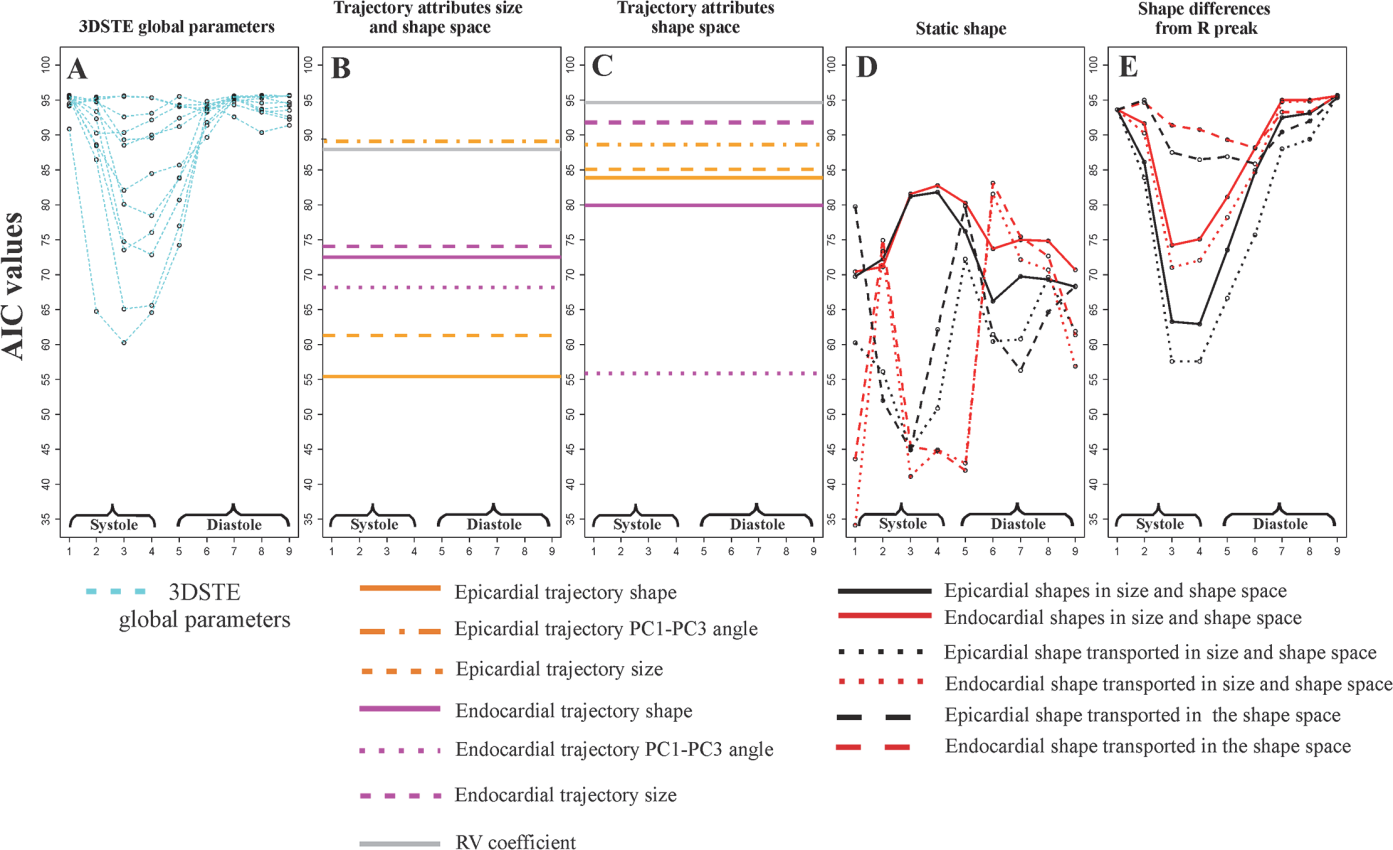
Results of logistic regressions using Control/HCM as a binary response and the entire bulk of morphological parameters used in this study. The y-axis always represents the Akaike Information Criterion value for any analysis, while the x-axis represents the nine homologous times. Smaller AICs indicate the best models. (A) STE global parameters; corresponding *p*-values can be found in Table 3. (B) Trajectory attributes in the size and shape space; as these attributes are proper of the entire shape trajectory they are not referred to any particular homologous time; corresponding *p*-values can be found in Table 5. (C) Trajectory attributes in the shape space: as these attributes are proper of the entire shape trajectory they are not referred to any particular homologous time; corresponding *p*-values can be found in Table 5. (D) LV static shape analyses using pure shapes, shape transported in the size and shape space and shapes transported in the shape space; corresponding *p*-values can be found in Table 4. (E) Shape differences from the R peak for the same types of parameters in D; corresponding *p*-values can be found in Table 4. Among static parameters we found that global longitudinal displacement is the best traditional descriptor at end systolic homologous time. Differences of pure epicardial shapes from R peak perform nearly in the same way. Static shapes (pure shapes in size and shape space, shapes transported in the size and space and shapes transported in the shape space), instead, are performing also in diastole. Endocardial shapes transported in both size and shape space and shape space show the lowest AICs. The courses of transported shapes and pure shapes follow inverse patterns. All trajectory attributes returned significant logistic regressions (Table 5) except for endocardial trajectory size and RV coefficient, both computed in the shape space.

Fig 7 illustrates the course of AIC values at homologous times for static parameters and trajectory attributes.

**Table 5:** Logistic regression AIC and *p*-values for trajectory attributes.

Size and shape space		Shape space	
Trajectory attributes	AIC	Trajectory attributes	AIC
Epicardial trajectory shape	55.41	Epicardial trajectory shape	83.84
Epicardial PC1-PC3 angle	89.53	Epicardial PC1-PC3 angle	88.65
Epicardial trajectory size	61.26	Epicardial trajectory size	85.11
Endocardial trajectory shape	72.43	Endocardial trajectory shape	79.88
Endocardial PC1-PC3 angle	68.19	Endocardial PC1-PC3 angle	55.88
Endocardial trajectory size	74.019	Endocardial trajectory size	91.84
RV coefficient	87.91	RV coefficient	94.22
**Corresponding *p*-values**		**Corresponding *p*-values**	
Epicardial trajectory shape	**0.0000**	Epicardial trajectory shape	**0.0013**
Epicardial PC1-PC3 angle	**0.0133**	Epicardial PC1-PC3 angle	**0.0080**
Epicardial trajectory size	**0.0000**	Epicardial trajectory size	**0.0010**
Endocardial trajectory shape	**0.0000**	Endocardial trajectory shape	**0.0002**
Endocardial PC1-PC3 angle	**0.0000**	Endocardial PC1-PC3 angle	**0.0000**
Endocardial trajectory size	**0.0000**	Endocardial trajectory size	0.0500
RV coefficient	**0.0054**	RV coefficient	0.2300

In bold significant results.

**Fig 8:**
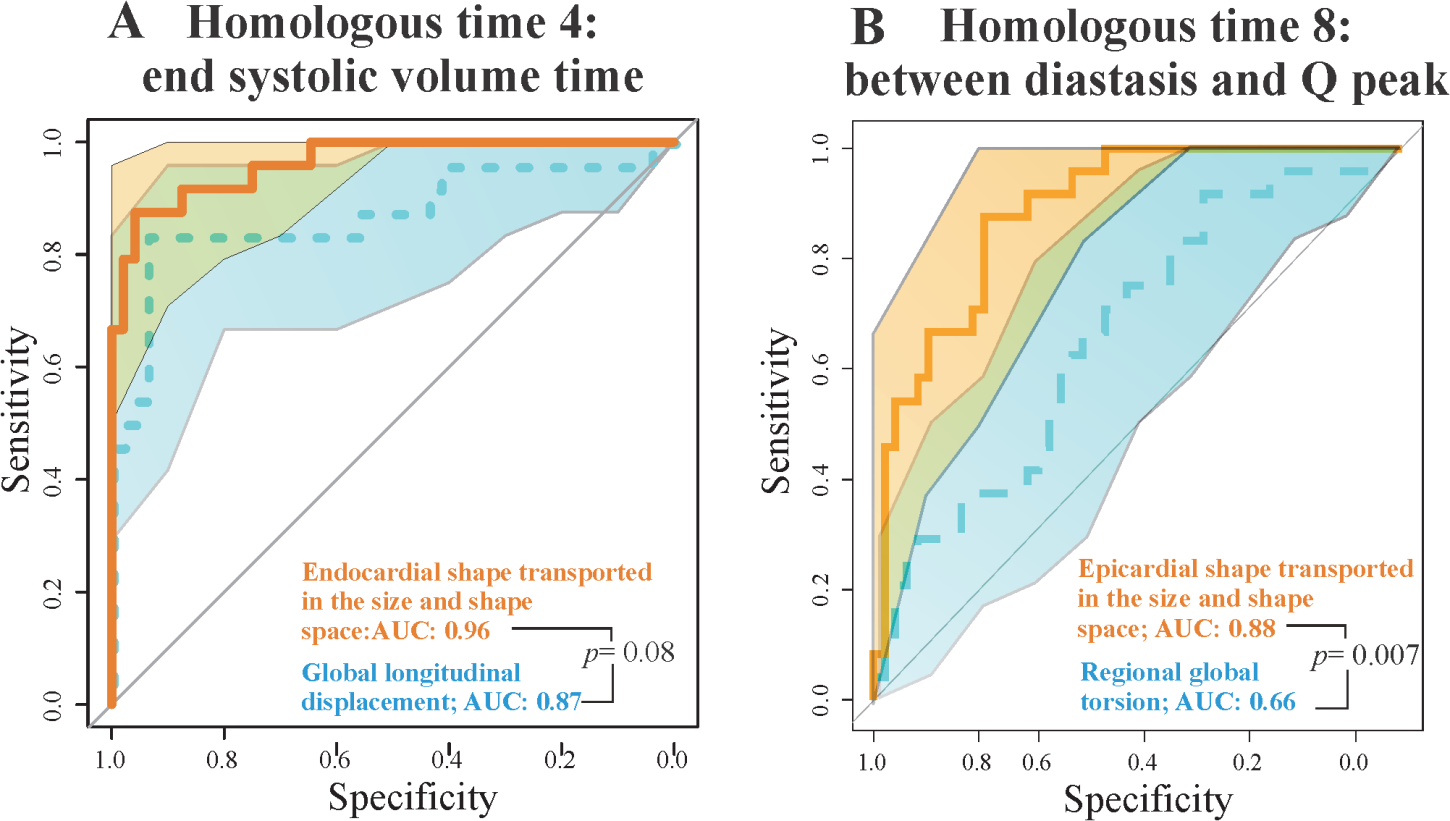
ROC curves for the best STE descriptors and our best static morphometric descriptors in systole and diastole. (A) Endocardial shape transported in the size and shape space *vs*. global longitudinal displacement. (B) Epicardial shape transported in the size and shape space *vs*. regional global torsion. Shaded areas represent the 95% of confidence interval for ROC curves. The *p*-values refer to the Delong test for differences in ROC prediction.

In particular, epicardial trajectory shape in the size and shape space and endocardial PC1-PC3 angle in the shape space show very low AIC values. We contrasted the best 3DSTE parameter in systole (Longitudinal displacement) and diastole (Regional global torsion) with the endocardial shape transported in the size and shape space and epicardial shape transported in the size and shape space respectively via ROC curves comparisons as shown in Fig 8.

**Fig 9:**
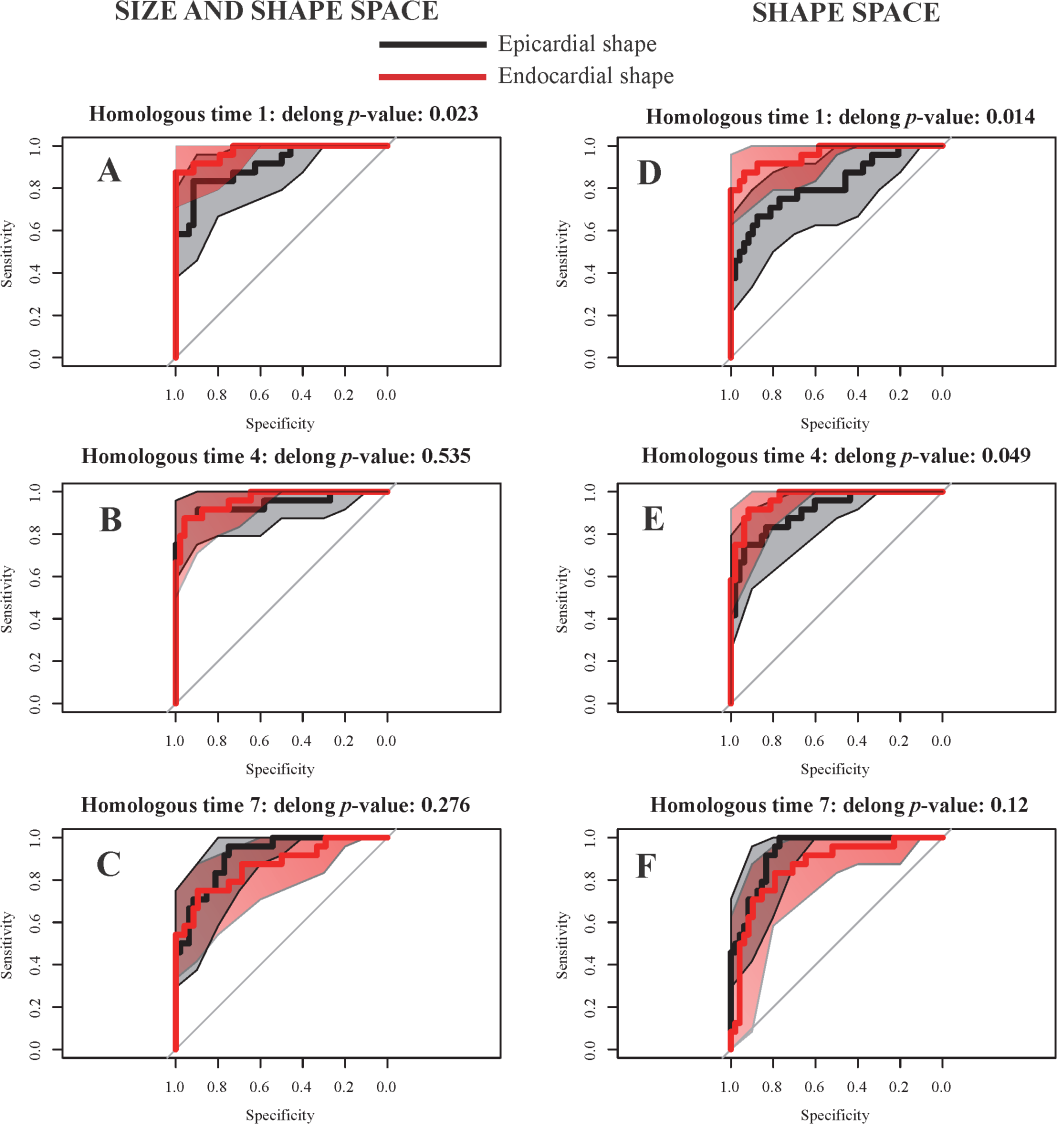
ROC curves for epicardium and endocardium transported in the size and shape space and in the shape space in three meaningful homologous times covering the entire cardiac revolution. (A-C) Size and shape space. (D-F) Shape space. Delong method was adopted to test differences in ROC curve prediction.

Geometric Morphometrics parameters have always larger AUCs. De Long et al. [25] non parametric approach suggests that for diastolic comparison the AUC difference is statistically significant, while the systolic one is very close to significance. Fig 9 shows the comparison between endocardium and epicardium at relevant homologous times. From R peak to the end systolic volume time, endocardium always predicts HCM better than epicardium, while in meso-diastole their ROC curves are not significantly different.

### Morphological Integration

**Fig 10:**
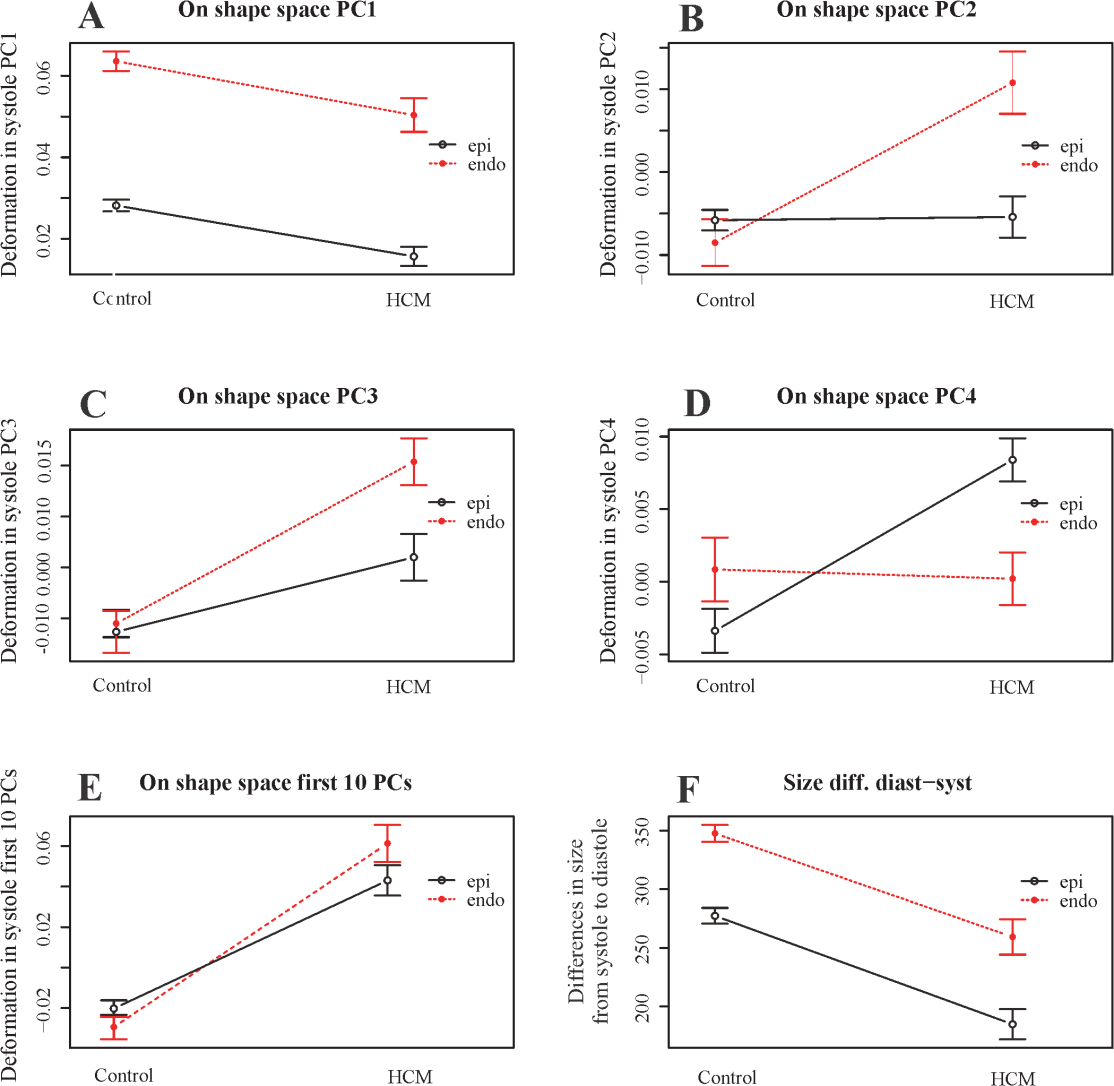
Two-way MANOVA (for shape) and ANOVA (for size) models. (A-D) Univariate interaction plots for the first 4 PCs of shape data transported in the shape space; these plot are aimed at showing that single PCs behave in different manner thus making necessary the use of a the multivariate test. (E) Just for sake of visualization we show the interaction plot where y-axis is represented by the Canonical Correlation scores coming from a multivariate model that includes the first 10 PCs as a response and Control/HCM as binary predictor; multivariate euclidean distances between the 4 categories (Control/HCM/Endocardium/Epicardium) can be found in Table 6. (F) Interaction plot for size.

As preliminary shown in Fig 6, RV coefficient between LV endocardium and epicardium during heart revolution is smaller in HCM than in control sample. ANOVA shows this difference as significant (*p*-value: 0.008). Results of the 2-way factorial design are shown in Fig 10.

**Table 6:** Multivariate Euclidean distances between first 10 PC scores of data transported in the shape space for the 2 way MANOVA design.

	Control Endocardium	HCM Endocardium	Control Epicardium	HCM Epicardium
Control Endocardium	0.000	0.029	0.036	0.049
HCM Endocardium	0.029	0.000	0.033	0.041
Control Epicardium	0.036	0.033	0.000	0.020
HCM Epicardium	0.049	0.041	0.020	0.000

We show the course of the first single 4 PC scores and the combination of first 10 PC scores; in this case, just for sake of visualization, the response variable in y-axis comes from a Canonical Correlation Analysis between first 10 PC scores of data shifted in the shape space corresponding to end systolic volume homologous time and the numeric factor Control/HCM. 2-way permutated MANOVA was performed using the entire set of multivariate response. Multivariate euclidean distances between the 2 categories (each with 2 levels) (Control/HCM/Endocardium/Epicardium) can be found in Table 6.

Interaction was found only for the data shifted on the shape space, while for size we observe parallel courses of Control/HCM*Epicardium/endocardium. This result suggests that pure-shape endocardial deformation in systole is larger than the epicardial one and that, passing from healthy to HCM individuals, the majority of differences are found on the shape changes of endocardium. Pure contraction of epicardium and endocardium, instead, scales equally in Control and HCM individuals, being in HCM significantly smaller as repeatedly reported in the Literature [4].

### The relationship between Geometric Morphometrics indicators and 3DSTE parameters

Correlations between the various Geometric Morphometrics indicators described above and original 3DSTE parameters are shown in S1 Table. Some indicators are correlated with classical 3DSTE variables, while some trajectory attributes, such as angles, show significant correlations with very few 3DSTE variables and with very weak correlation.

### Static shape analysis at homologous time

**Fig 11:**
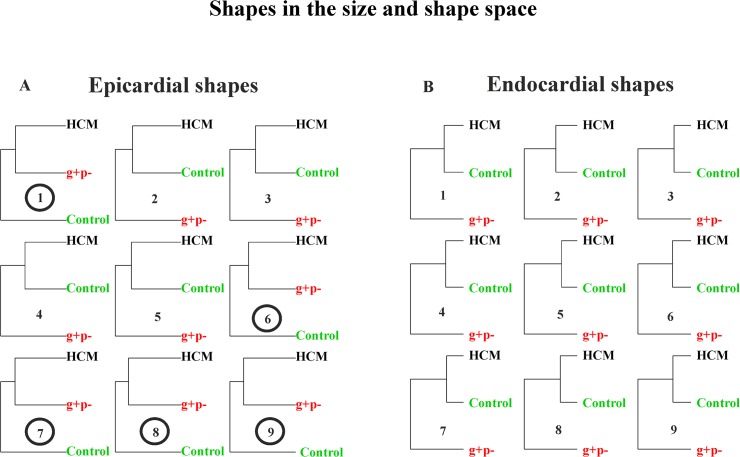
UPGMA analyses performed at the nine homologous times on pure shapes. (A) Endocardial shapes. (B) Epicardial shapes. Circles represent the analysis where g+p- individuals cluster together with HCM patients.

Fig 11 shows the UPGMA analyses using the first 5 PCs for any static GPA+PCA (size and shape space) performed at homologous times.

**Fig 12:**
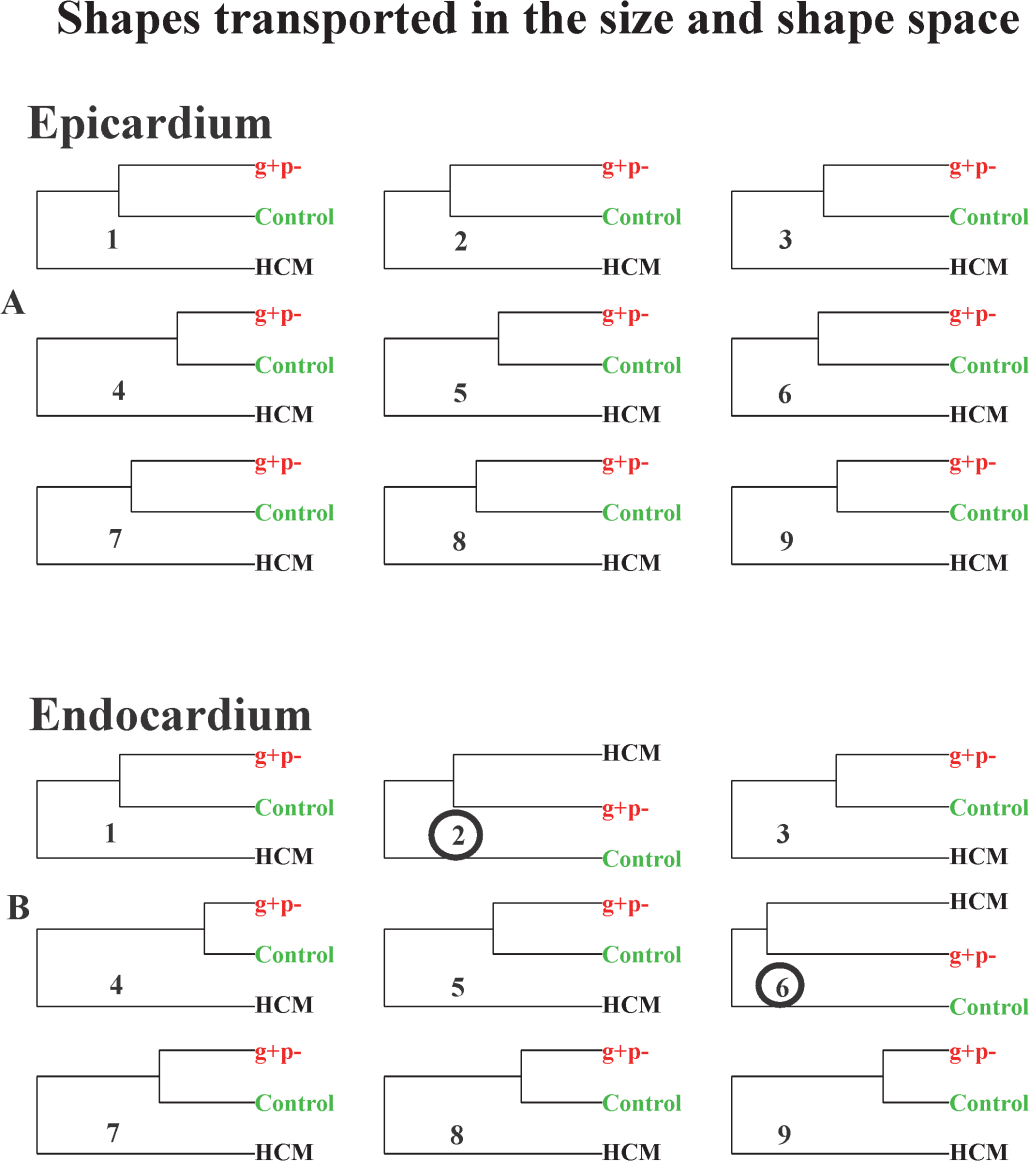
UPGMA analyses performed at the nine homologous times on shapes transported on the size and shape space. (A) Epicardial shapes. (B) Endocardial shapes. Circles represent the analysis where g+p- individuals cluster together with HCM patients.

**Fig 13:**
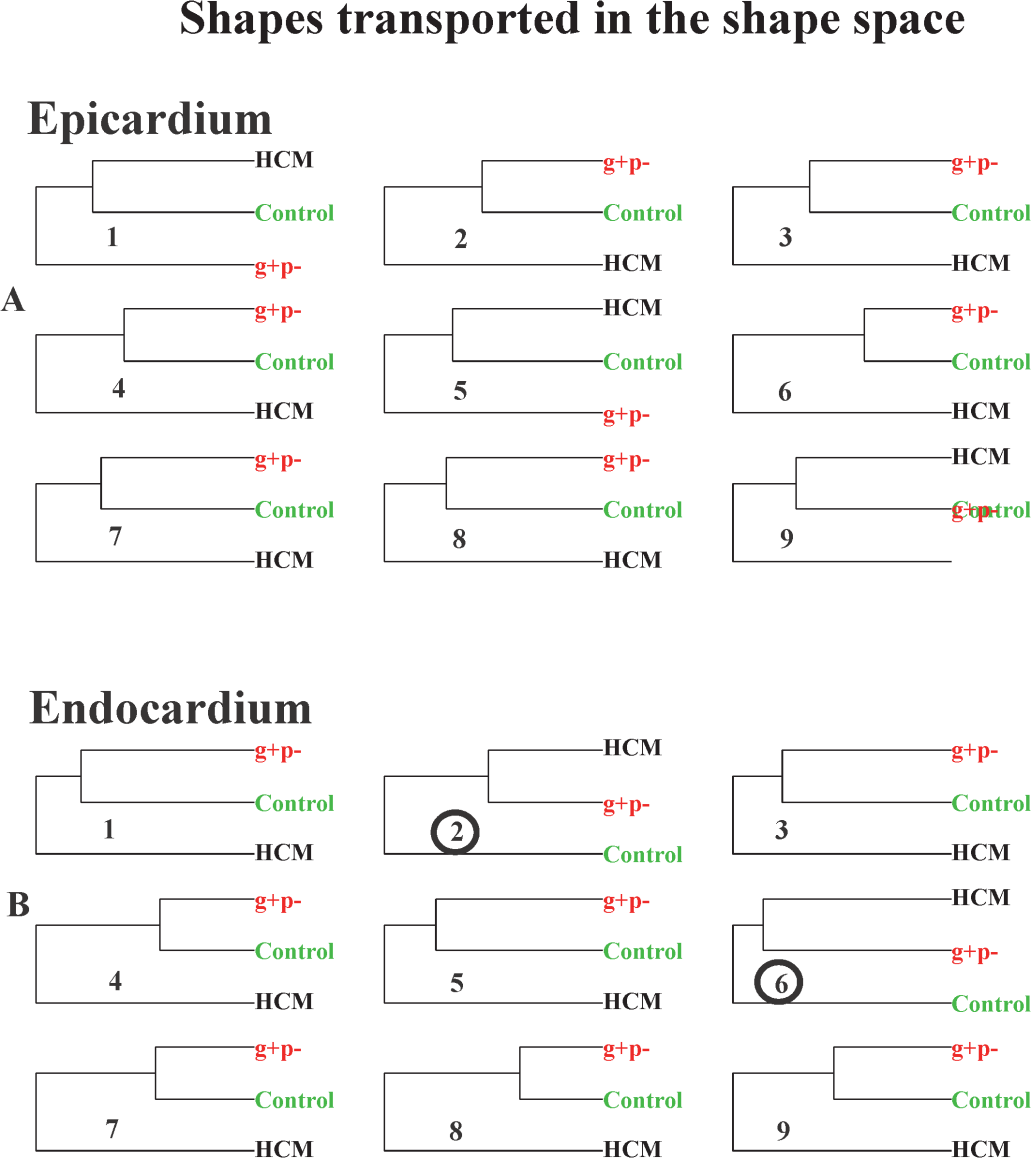
UPGMA analyses performed at the nine homologous times on shapes transported on the shape space. (A) Epicardial shapes. (B) Endocardial shapes. Circles represent the analysis where g+p- individuals cluster together with HCM patients.

It is evident how in homologous times 3^rd^, 4^th^ and 5^th^ g+p- individuals clearly approach HCM patients. Fig 12 and Fig 13 show the same analysis performed on data transported in the size and shape space and in the shape space. In this case g+p- approach HCM patients in the endocardial deformation, in particular in meso-systole and meso-diastole.

## Discussion

The results obtained in the present study insert in the broad range of investigations regarding deformation in LV of HCM patients. We feel, however, that a new look to the traditional concept of deformation, as applied in cardiovascular sciences, could help in identifying new potential indicators for diastolic dysfunction or for incumbent pathology. Geometric Morphometrics represents an unexplored way to process echocardiographic data with the aim of unveiling LV true shape deformation during cardiac morphological revolution. First, we stress that a deformation should be always evaluated *per se*. Thus, the shape to which it applies should be disregarded if the goal is analyzing the intrinsic deformative process. To do that, the linear shift is a suitable procedure for transporting a set of deformations toward a common shape. Transporting separately endocardium and epicardium, leads to a finer appreciation of their differential fate during heart cycle. Some studies exist that analyzed the two layer separately [27–30]. These investigations highlight that epicardium and endocardium are better interpreted in terms of deformation if evaluated independently. The same conclusion is supported here. Our approach allows evaluating the shape of the trajectories themselves that are expression of function. Traditional 3DSTE indicators are usually computed as differences from the R peak. This makes the study of diastolic dysfunction difficult as, approaching to the R peak, differences approach to 0 by definition. Moreover, these 3DSTE parameters do not discriminate between endocardium and epicardium behaviour. On the opposite, the use of Geometric Morphometrics combined with the notion of homologous times, permits evaluating the diastolic phase in a new manner because data are transported toward the Grand Mean that, in the context of LV cycle, can be thought of as the mean shape between LV shapes at meso-systolic and meso-diastolic phases. Transporting deformation, i.e. subtracting local means to any individual LV cycle and adding the Grand Mean, means that even end diastolic phase is considered a true deformed state whereas usually, the sole end systolic time is interpreted in this way. All these computations should be performed on endocardium and epicardium separately. In fact, their trajectories are very different in both size and shape space and in the shape space. This can be appreciated in Fig 3 and Fig 5 as well as upon the significant difference for trajectories attributes existing between Control and HCM individuals (as shown in Table 2). In particular, the endocardium shows very different PC1-PC3 angle that points in visibly opposite directions in the 2 groups. This parameter is highly discriminating having an AIC much smaller of that of traditional 3DSTE parameters associated to logistic regression (Fig 7). Besides orientation, the shape of epicardial and endocardial trajectories are very different in both size and shape space and shape space in Control and HCM individuals as depicted in Fig 5. HCM have much smaller (PC1) and much less rounded (PC2 and PC3) trajectories in comparison to controls. The shape of epicardial trajectory in size and shape space possesses a very low AIC of logistic regression. This is certainly due to the fact that the smaller contraction in HCM patients is detected by the shape of epicardial trajectory in the size and shape space.

Separating epicardium and endocardium allows us appreciating their integration during LV cycle: HCM patients show a significantly smaller morphological integration (relatively to Control) solely in the size and shape space. This means that their changes across the 9 homologous times loose the normal covariation condition due to an abnormal contraction. In the shape space this signal is not present. ROC curves clearly show that our morphometric indicators perform better than traditional 3DSTE parameters (Fig 8). This is particularly true in diastole because, as stated before, this phase is recognized by the linear shift procedure as a true deformed state. ROC curves of epicardium and endocardium show that endocardium predicts pathology significantly better in systole in both size and shape space and shape space. This is also evident by our 2-way MANOVA design performed on the shape space in systole: Control and HCM individuals differ more for endocardium than for epicardium, while size scales equally. This result could be used to speculate about the role of the muscular disarray affecting HCM individuals: while the functional impairment of size equally affects epicardium and endocardium, the anomalies on pure shape are more evident in the endocardium. This means that the endocardium, when passing from control to HCM conditions, shows a more severely impaired systolic deformation in terms of pure shape in comparison to epicardium. Of course this does not inform us about the location of muscular disarray: this information inevitably should come from histo-pathological investigations [31]. However, the consequences of this disarray could be evaluated upon the results we obtained here. Recently Martinez Lagazpi et al. [32] investigated the role of vortex ring formation in LV filling for HCM pathology. They found that in HCM patients the percentage of LV filling due to vortex formation is significantly reduced in comparison to healthy subjects. They related this evidence with chamber sphericity. This correlation between shape and vortex formation in diastole is highly suggestive here because pure shapes in size and shape space discriminate HCM better in diastole than in systole. This could be interpreted as an evidence that systolic contraction “tries” to achieve a normal shape due to the function that LV must fulfil irrespective of the pathological condition, while in diastole the shape differences are more settling. When looking at transported data (in either size and shape space or shape space) systole becomes more discriminating (Fig 7) because these kinds of data represent a deformation not just a shape.

It is worth noting that our best trajectory indicator (i.e. PC1-PC3 angle for endocardial data transported in the shape space), illustrated in Fig 7 and in S4 Fig, is significantly related only with 3 out of 12 variables, having on top a weak correlation. This means that this parameter represents something that is not captured by 3DSTE variables. The same holds true for other indicators such as RV coefficient and PC1 of epicardial and endocardial shapes in the size and shape space. On the other hand, other indicators correlate better with 3DSTE parameters and this is expected in general terms since 3DSTE traditional parameters are calculated from the same landmark clouds we used for our size and shape analyses. The fact that a trajectory attribute correlates less may thus indicate that a genuine 4D approach could capture emergent properties hardly derived from the calculation of any parameter evaluated at one single time frame. Therefore, the information provided by the morphometric analyses might be considered relatively independent from that given by 3DSTE traditional parameters and as such deserving a special independent attention and study.

The preliminary inclusion of the 3 g+p- individuals deserves particular attention: using pure shapes in the size and shape space they approach HCM in 5/9 homologous times for the epicardial shape mainly in diastole (Fig 11). This is particularly important because diastolic dysfunction was hypothesized for g+p- individuals as evidence of incumbent pathology [33]. When the deformation is investigated by using transported data, instead, the g+p- individuals cluster with HCM patients (Fig 12 and Fig 13) for endocardial deformation in meso-systolic and meso-diastolic phases. These evidences suggest that this type of investigation should be pursued using a larger sample of g+p- individuals because the possibility to predict genotype from phenotype could help both prevention and treatment and could be far reaching.

**Fig 14:**
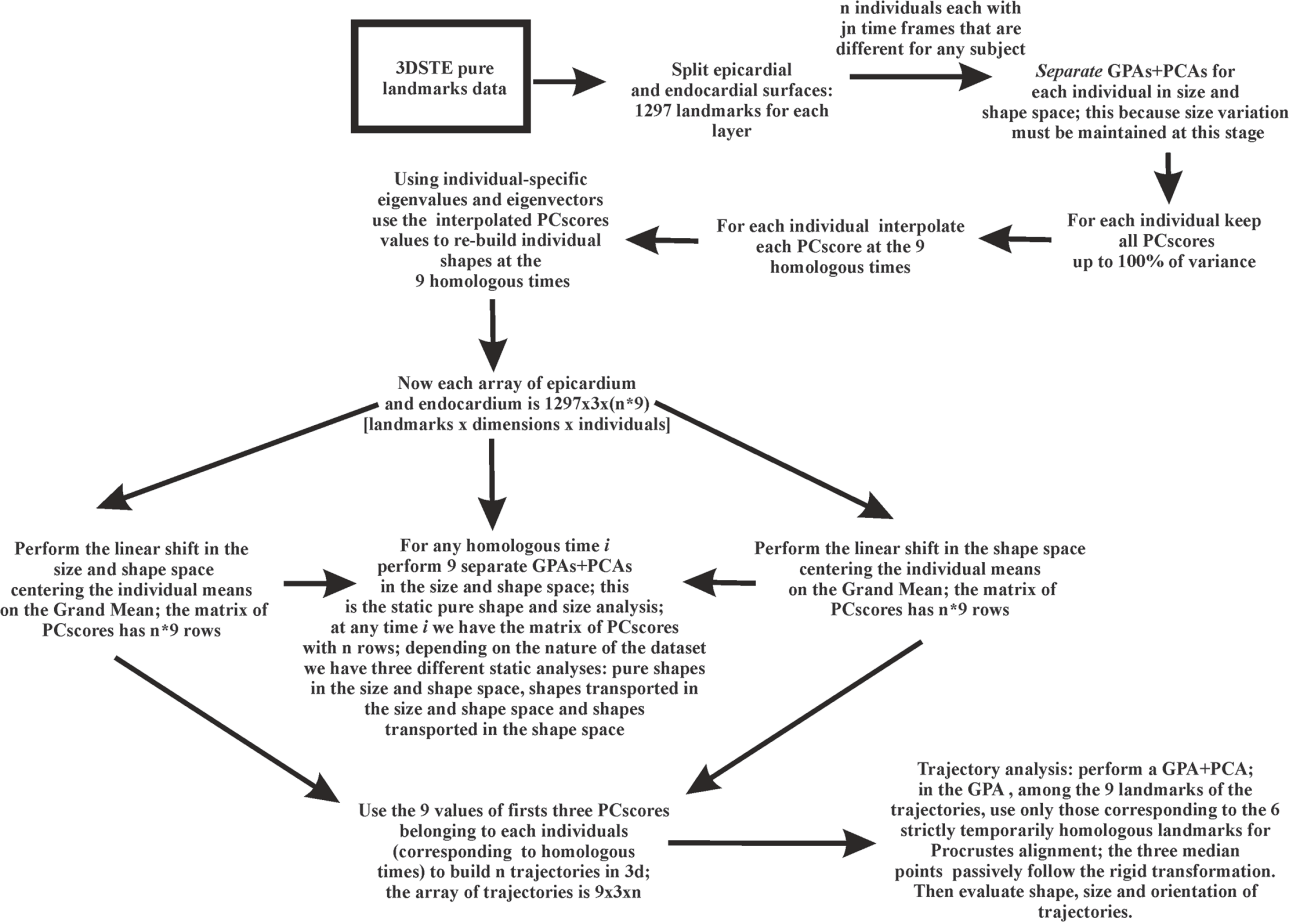
Step by step flowchart of the entire procedure presented in the paper.

It might be argued that a great deal of manual operations are needed to replicate our results which is true at present since the software normally equipping Artida devices is not open source being copyrighted by Toshiba. As a consequence the landmark cloud is not visible normally. On the other hand, it could be easy to implement that software by including the functionalities illustrated in the conceptual flowchart depicted in Fig 14. It is foreseen that, developing further the interest in morphometrics studies, the possible implementations looked for above might soon become ready.

## Supporting Information

S1 Fig Trajectory PC1 PC2 PC3 of transported data of epicardium in the size and shape space. Animated GIFs of trajectory shapes and morphologies associated to the first three PC scores in both size and shape space and shape space. In green healthy subjects, in black HCM individuals; numbers in the trajectories animations refer to the sequential 9 homologous times. S1 Table reports orrelations between morphometric indicators described in the paper and traditional 3DSTE global parameters.
(GIF)

S2 Fig Trajectory PC1 PC2 PC3 of transported data of epicardium in the shape space. Animated GIFs of trajectory shapes and morphologies associated to the first three PC scores in both size and shape space and shape space. In green healthy subjects, in black HCM individuals; numbers in the trajectories animations refer to the sequential 9 homologous times. S1 Table reports orrelations between morphometric indicators described in the paper and traditional 3DSTE global parameters.
(GIF)

S3 Fig Trajectory PC1 PC2 PC3 of transported data of endocardium in the size and shape space. Animated GIFs of trajectory shapes and morphologies associated to the first three PC scores in both size and shape space and shape space. In green healthy subjects, in black HCM individuals; numbers in the trajectories animations refer to the sequential 9 homologous times. S1 Table reports orrelations between morphometric indicators described in the paper and traditional 3DSTE global parameters.
(GIF)

S4 Fig Trajectory PC1 PC2 PC3 of transported data of endocardium in the shape space. Animated GIFs of trajectory shapes and morphologies associated to the first three PC scores in both size and shape space and shape space. In green healthy subjects, in black HCM individuals; numbers in the trajectories animations refer to the sequential 9 homologous times. S1 Table reports orrelations between morphometric indicators described in the paper and traditional 3DSTE global parameters.
(GIF)

S5 Fig PC1 Control and HCM transported data of endocardium in the size and shape space. Animated GIFs of trajectory shapes and morphologies associated to the first three PC scores in both size and shape space and shape space. In green healthy subjects, in black HCM individuals; numbers in the trajectories animations refer to the sequential 9 homologous times. S1 Table reports orrelations between morphometric indicators described in the paper and traditional 3DSTE global parameters.
(GIF)

S6 Fig PC1 Control and HCM transported data of endocardium in the shape space. Animated GIFs of trajectory shapes and morphologies associated to the first three PC scores in both size and shape space and shape space. In green healthy subjects, in black HCM individuals; numbers in the trajectories animations refer to the sequential 9 homologous times. S1 Table reports orrelations between morphometric indicators described in the paper and traditional 3DSTE global parameters.
(GIF)

S7 Fig PC1 Control and HCM transported data of epicardium in the size and shape space. Animated GIFs of trajectory shapes and morphologies associated to the first three PC scores in both size and shape space and shape space. In green healthy subjects, in black HCM individuals; numbers in the trajectories animations refer to the sequential 9 homologous times. S1 Table reports orrelations between morphometric indicators described in the paper and traditional 3DSTE global parameters.
(GIF)

S8 Fig PC1 Control and HCM transported data of epicardium in the shape space. Animated GIFs of trajectory shapes and morphologies associated to the first three PC scores in both size and shape space and shape space. In green healthy subjects, in black HCM individuals; numbers in the trajectories animations refer to the sequential 9 homologous times. S1 Table reports orrelations between morphometric indicators described in the paper and traditional 3DSTE global parameters.
(GIF)

S9 Fig PC2 Control and HCM transported data of endocardium in the size and shape space. Animated GIFs of trajectory shapes and morphologies associated to the first three PC scores in both size and shape space and shape space. In green healthy subjects, in black HCM individuals; numbers in the trajectories animations refer to the sequential 9 homologous times. S1 Table reports orrelations between morphometric indicators described in the paper and traditional 3DSTE global parameters.
(GIF)

S10 Fig PC2 Control and HCM transported data of endocardium in the shape space. Animated GIFs of trajectory shapes and morphologies associated to the first three PC scores in both size and shape space and shape space. In green healthy subjects, in black HCM individuals; numbers in the trajectories animations refer to the sequential 9 homologous times. S1 Table reports orrelations between morphometric indicators described in the paper and traditional 3DSTE global parameters.
(GIF)

S11 Fig PC2 Control and HCM transported data of epicardium in the size and shape space. Animated GIFs of trajectory shapes and morphologies associated to the first three PC scores in both size and shape space and shape space. In green healthy subjects, in black HCM individuals; numbers in the trajectories animations refer to the sequential 9 homologous times. S1 Table reports orrelations between morphometric indicators described in the paper and traditional 3DSTE global parameters.
(GIF)

S12 Fig PC2 Control and HCM transported data of epicardium in the shape space. Animated GIFs of trajectory shapes and morphologies associated to the first three PC scores in both size and shape space and shape space. In green healthy subjects, in black HCM individuals; numbers in the trajectories animations refer to the sequential 9 homologous times. S1 Table reports orrelations between morphometric indicators described in the paper and traditional 3DSTE global parameters.
(GIF)

S13 Fig PC3 Control and HCM transported data of endocardium in the size shape space. Animated GIFs of trajectory shapes and morphologies associated to the first three PC scores in both size and shape space and shape space. In green healthy subjects, in black HCM individuals; numbers in the trajectories animations refer to the sequential 9 homologous times. S1 Table reports orrelations between morphometric indicators described in the paper and traditional 3DSTE global parameters.
(GIF)

S14 Fig PC3 Control and HCM transported data of endocardium in the shape space. Animated GIFs of trajectory shapes and morphologies associated to the first three PC scores in both size and shape space and shape space. In green healthy subjects, in black HCM individuals; numbers in the trajectories animations refer to the sequential 9 homologous times. S1 Table reports orrelations between morphometric indicators described in the paper and traditional 3DSTE global parameters.
(GIF)

S15 Fig PC3 Control and HCM transported data of epicardium in the size and shape space. Animated GIFs of trajectory shapes and morphologies associated to the first three PC scores in both size and shape space and shape space. In green healthy subjects, in black HCM individuals; numbers in the trajectories animations refer to the sequential 9 homologous times. S1 Table reports orrelations between morphometric indicators described in the paper and traditional 3DSTE global parameters.
(GIF)

S16 Fig PC3 Control and HCM transported data of epicardium in the shape space. Animated GIFs of trajectory shapes and morphologies associated to the first three PC scores in both size and shape space and shape space. In green healthy subjects, in black HCM individuals; numbers in the trajectories animations refer to the sequential 9 homologous times. S1 Table reports orrelations between morphometric indicators described in the paper and traditional 3DSTE global parameters.
(GIF)

S17 Fig PC1 PC2 PC3 Control and HCM transported data of endocardium in the size and shape space. Animated GIFs of trajectory shapes and morphologies associated to the first three PC scores in both size and shape space and shape space. In green healthy subjects, in black HCM individuals; numbers in the trajectories animations refer to the sequential 9 homologous times. S1 Table reports orrelations between morphometric indicators described in the paper and traditional 3DSTE global parameters.
(GIF)

S18 Fig PC1 PC2 PC3 Control and HCM transported data of endocardium in the shape space. Animated GIFs of trajectory shapes and morphologies associated to the first three PC scores in both size and shape space and shape space. In green healthy subjects, in black HCM individuals; numbers in the trajectories animations refer to the sequential 9 homologous times. S1 Table reports orrelations between morphometric indicators described in the paper and traditional 3DSTE global parameters.
(GIF)

S19 Fig PC1 PC2 PC3 Control and HCM transported data epicardium in the size and shape space. Animated GIFs of trajectory shapes and morphologies associated to the first three PC scores in both size and shape space and shape space. In green healthy subjects, in black HCM individuals; numbers in the trajectories animations refer to the sequential 9 homologous times. S1 Table reports orrelations between morphometric indicators described in the paper and traditional 3DSTE global parameters.
(GIF)

S20 Fig PC1 PC2 PC3 Control and HCM transported data of epicardium in the shape space. Animated GIFs of trajectory shapes and morphologies associated to the first three PC scores in both size and shape space and shape space. In green healthy subjects, in black HCM individuals; numbers in the trajectories animations refer to the sequential 9 homologous times. S1 Table reports orrelations between morphometric indicators described in the paper and traditional 3DSTE global parameters.
(GIF)

S1 Table Correlations between morphometric indicators described in the paper and traditional 3DSTE global parameters. In bold significant results.
(DOC)
