# Supplementary figures and images for: A New 4D Trajectory-Based Approach Unveils Abnormal LV Revolution Dynamics in Hypertrophic Cardiomyopathy
Source: PLoS One. 2015 Apr 13;10(4):e0122376. doi: 10.1371/journal.pone.0122376 (PMC4395437; doi:10.1371/journal.pone.0122376)

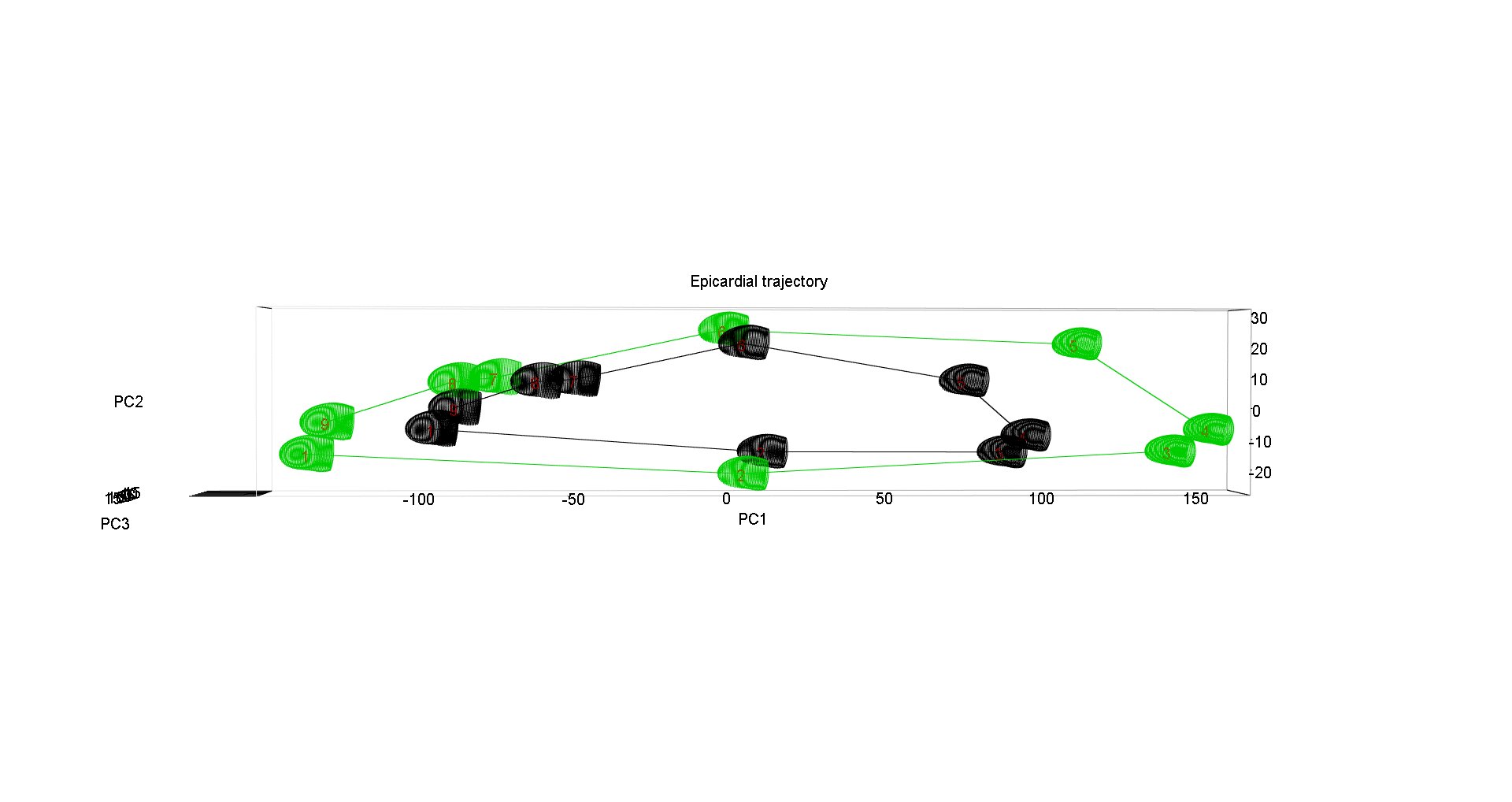

Supplement: S1 Fig — Animated GIFs of trajectory shapes and morphologies associated to the first three PC scores in both size and shape space and shape space. In green healthy subjects, in black HCM individuals; numbers in the trajectories animations refer to the sequential 9 homologous times. S1 Table reports orrelations between morphometric indicators described in the paper and traditional 3DSTE global parameters. (GIF) [file pone.0122376.s001.gif]

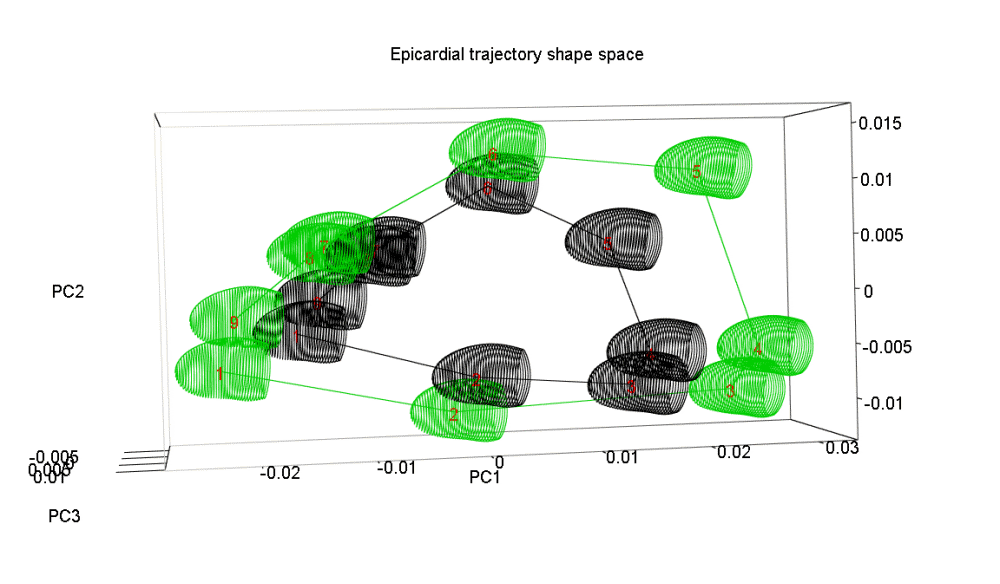

Supplement: S2 Fig — Animated GIFs of trajectory shapes and morphologies associated to the first three PC scores in both size and shape space and shape space. In green healthy subjects, in black HCM individuals; numbers in the trajectories animations refer to the sequential 9 homologous times. S1 Table reports orrelations between morphometric indicators described in the paper and traditional 3DSTE global parameters. (GIF) [file pone.0122376.s002.gif]

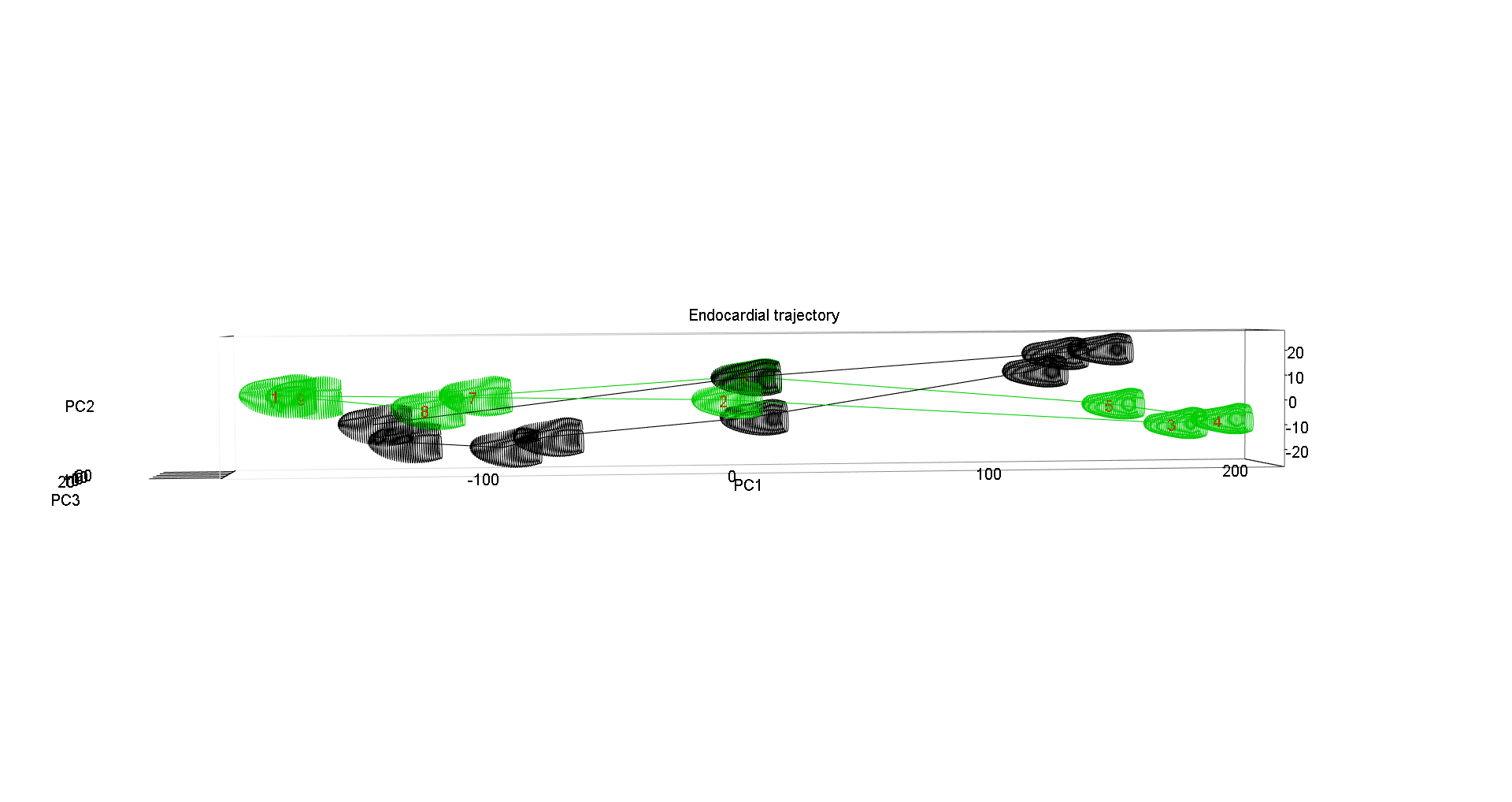

Supplement: S3 Fig — Animated GIFs of trajectory shapes and morphologies associated to the first three PC scores in both size and shape space and shape space. In green healthy subjects, in black HCM individuals; numbers in the trajectories animations refer to the sequential 9 homologous times. S1 Table reports orrelations between morphometric indicators described in the paper and traditional 3DSTE global parameters. (GIF) [file pone.0122376.s003.gif]

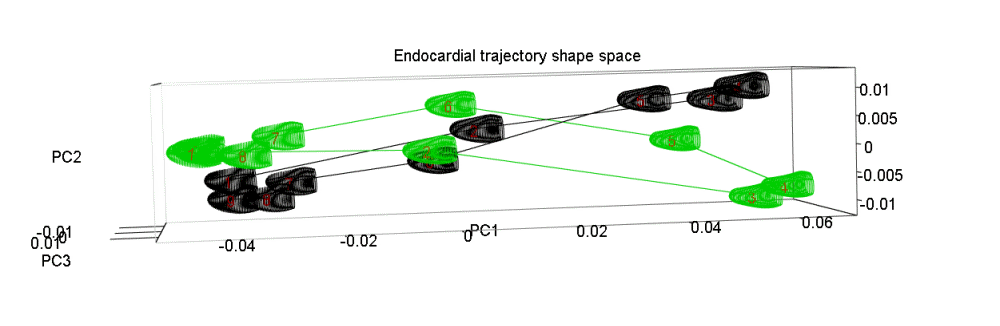

Supplement: S4 Fig — Animated GIFs of trajectory shapes and morphologies associated to the first three PC scores in both size and shape space and shape space. In green healthy subjects, in black HCM individuals; numbers in the trajectories animations refer to the sequential 9 homologous times. S1 Table reports orrelations between morphometric indicators described in the paper and traditional 3DSTE global parameters. (GIF) [file pone.0122376.s004.gif]

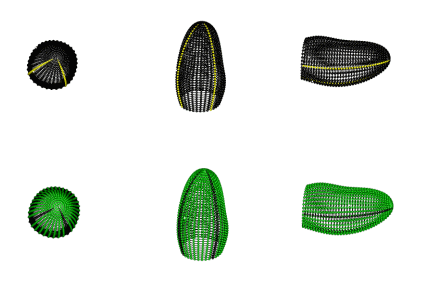

Supplement: S5 Fig — Animated GIFs of trajectory shapes and morphologies associated to the first three PC scores in both size and shape space and shape space. In green healthy subjects, in black HCM individuals; numbers in the trajectories animations refer to the sequential 9 homologous times. S1 Table reports orrelations between morphometric indicators described in the paper and traditional 3DSTE global parameters. (GIF) [file pone.0122376.s005.gif]

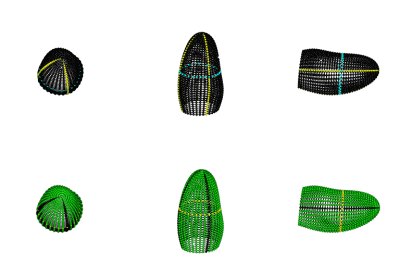

Supplement: S6 Fig — Animated GIFs of trajectory shapes and morphologies associated to the first three PC scores in both size and shape space and shape space. In green healthy subjects, in black HCM individuals; numbers in the trajectories animations refer to the sequential 9 homologous times. S1 Table reports orrelations between morphometric indicators described in the paper and traditional 3DSTE global parameters. (GIF) [file pone.0122376.s006.gif]

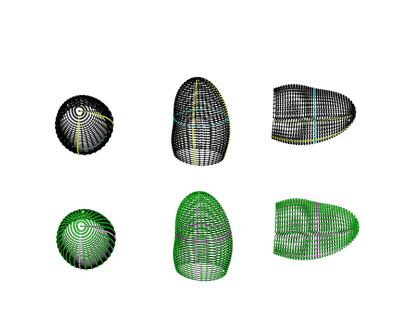

Supplement: S7 Fig — Animated GIFs of trajectory shapes and morphologies associated to the first three PC scores in both size and shape space and shape space. In green healthy subjects, in black HCM individuals; numbers in the trajectories animations refer to the sequential 9 homologous times. S1 Table reports orrelations between morphometric indicators described in the paper and traditional 3DSTE global parameters. (GIF) [file pone.0122376.s007.gif]

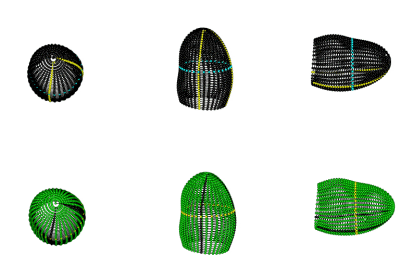

Supplement: S8 Fig — Animated GIFs of trajectory shapes and morphologies associated to the first three PC scores in both size and shape space and shape space. In green healthy subjects, in black HCM individuals; numbers in the trajectories animations refer to the sequential 9 homologous times. S1 Table reports orrelations between morphometric indicators described in the paper and traditional 3DSTE global parameters. (GIF) [file pone.0122376.s008.gif]

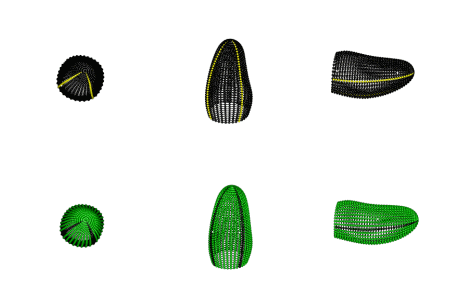

Supplement: S9 Fig — Animated GIFs of trajectory shapes and morphologies associated to the first three PC scores in both size and shape space and shape space. In green healthy subjects, in black HCM individuals; numbers in the trajectories animations refer to the sequential 9 homologous times. S1 Table reports orrelations between morphometric indicators described in the paper and traditional 3DSTE global parameters. (GIF) [file pone.0122376.s009.gif]

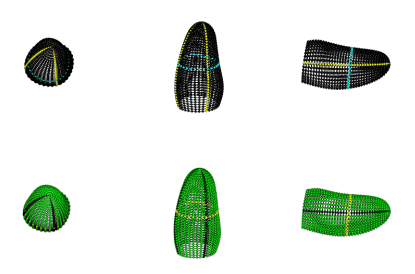

Supplement: S10 Fig — Animated GIFs of trajectory shapes and morphologies associated to the first three PC scores in both size and shape space and shape space. In green healthy subjects, in black HCM individuals; numbers in the trajectories animations refer to the sequential 9 homologous times. S1 Table reports orrelations between morphometric indicators described in the paper and traditional 3DSTE global parameters. (GIF) [file pone.0122376.s010.gif]

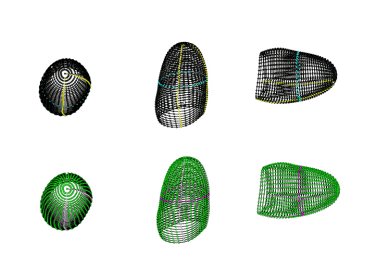

Supplement: S11 Fig — Animated GIFs of trajectory shapes and morphologies associated to the first three PC scores in both size and shape space and shape space. In green healthy subjects, in black HCM individuals; numbers in the trajectories animations refer to the sequential 9 homologous times. S1 Table reports orrelations between morphometric indicators described in the paper and traditional 3DSTE global parameters. (GIF) [file pone.0122376.s011.gif]

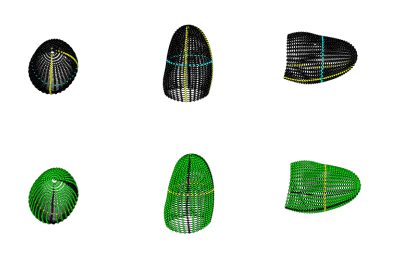

Supplement: S12 Fig — Animated GIFs of trajectory shapes and morphologies associated to the first three PC scores in both size and shape space and shape space. In green healthy subjects, in black HCM individuals; numbers in the trajectories animations refer to the sequential 9 homologous times. S1 Table reports orrelations between morphometric indicators described in the paper and traditional 3DSTE global parameters. (GIF) [file pone.0122376.s012.gif]

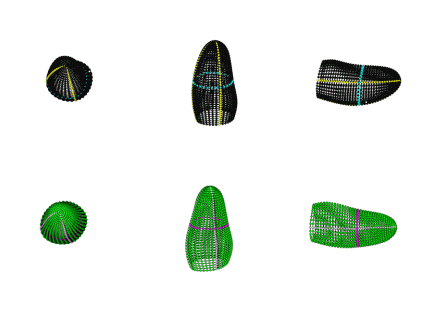

Supplement: S13 Fig — Animated GIFs of trajectory shapes and morphologies associated to the first three PC scores in both size and shape space and shape space. In green healthy subjects, in black HCM individuals; numbers in the trajectories animations refer to the sequential 9 homologous times. S1 Table reports orrelations between morphometric indicators described in the paper and traditional 3DSTE global parameters. (GIF) [file pone.0122376.s013.gif]

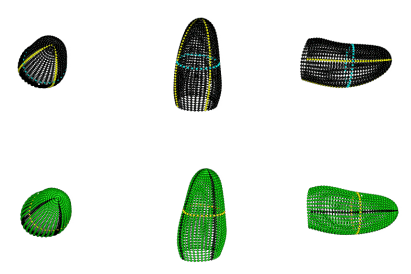

Supplement: S14 Fig — Animated GIFs of trajectory shapes and morphologies associated to the first three PC scores in both size and shape space and shape space. In green healthy subjects, in black HCM individuals; numbers in the trajectories animations refer to the sequential 9 homologous times. S1 Table reports orrelations between morphometric indicators described in the paper and traditional 3DSTE global parameters. (GIF) [file pone.0122376.s014.gif]

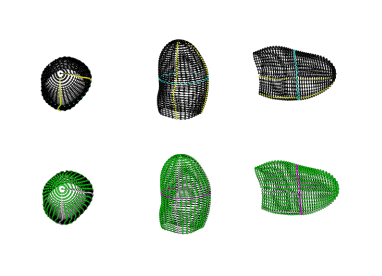

Supplement: S15 Fig — Animated GIFs of trajectory shapes and morphologies associated to the first three PC scores in both size and shape space and shape space. In green healthy subjects, in black HCM individuals; numbers in the trajectories animations refer to the sequential 9 homologous times. S1 Table reports orrelations between morphometric indicators described in the paper and traditional 3DSTE global parameters. (GIF) [file pone.0122376.s015.gif]

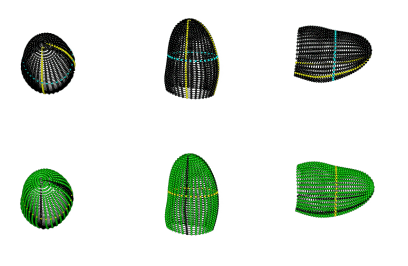

Supplement: S16 Fig — Animated GIFs of trajectory shapes and morphologies associated to the first three PC scores in both size and shape space and shape space. In green healthy subjects, in black HCM individuals; numbers in the trajectories animations refer to the sequential 9 homologous times. S1 Table reports orrelations between morphometric indicators described in the paper and traditional 3DSTE global parameters. (GIF) [file pone.0122376.s016.gif]

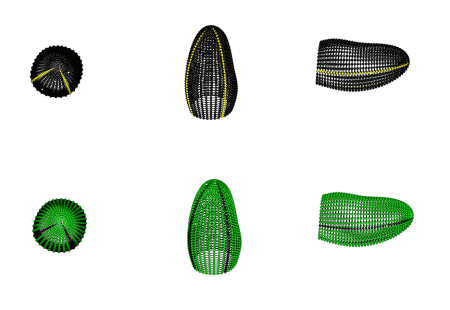

Supplement: S17 Fig — Animated GIFs of trajectory shapes and morphologies associated to the first three PC scores in both size and shape space and shape space. In green healthy subjects, in black HCM individuals; numbers in the trajectories animations refer to the sequential 9 homologous times. S1 Table reports orrelations between morphometric indicators described in the paper and traditional 3DSTE global parameters. (GIF) [file pone.0122376.s017.gif]

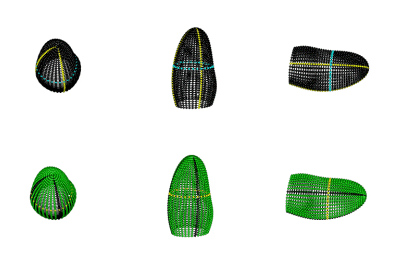

Supplement: S18 Fig — Animated GIFs of trajectory shapes and morphologies associated to the first three PC scores in both size and shape space and shape space. In green healthy subjects, in black HCM individuals; numbers in the trajectories animations refer to the sequential 9 homologous times. S1 Table reports orrelations between morphometric indicators described in the paper and traditional 3DSTE global parameters. (GIF) [file pone.0122376.s018.gif]

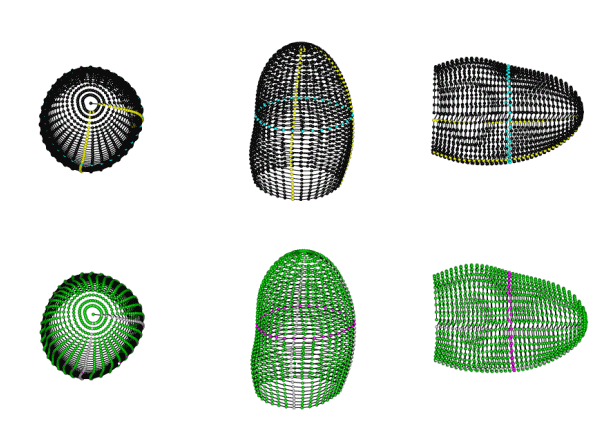

Supplement: S19 Fig — Animated GIFs of trajectory shapes and morphologies associated to the first three PC scores in both size and shape space and shape space. In green healthy subjects, in black HCM individuals; numbers in the trajectories animations refer to the sequential 9 homologous times. S1 Table reports orrelations between morphometric indicators described in the paper and traditional 3DSTE global parameters. (GIF) [file pone.0122376.s019.gif]

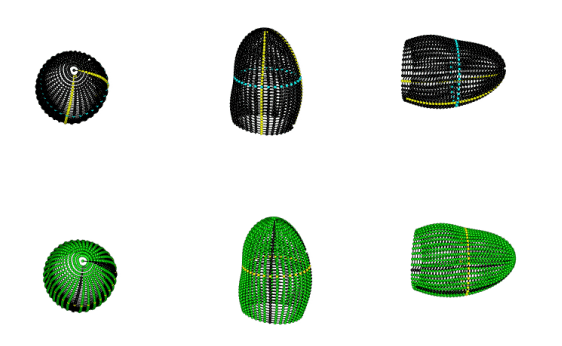

Supplement: S20 Fig — Animated GIFs of trajectory shapes and morphologies associated to the first three PC scores in both size and shape space and shape space. In green healthy subjects, in black HCM individuals; numbers in the trajectories animations refer to the sequential 9 homologous times. S1 Table reports orrelations between morphometric indicators described in the paper and traditional 3DSTE global parameters. (GIF) [file pone.0122376.s020.gif]
