# Supplementary material for: A New 4D Trajectory-Based Approach Unveils Abnormal LV Revolution Dynamics in Hypertrophic Cardiomyopathy
Source: PLoS One. 2015 Apr 13;10(4):e0122376. doi: 10.1371/journal.pone.0122376 (PMC4395437; doi:10.1371/journal.pone.0122376)
Supplement: S1 Table — In bold significant results. (DOC) [file pone.0122376.s021.doc]

| **Supplementary Table S1. Correlations between morphometric indicators described in the paper and traditional 3DSTE global parameters. In bold significant results.** | RStrain_global | CStrain_global | LStrain_global | Rotation_global | Twist_global | Torsion_Regional_global | Torsion_Basal_global | Strain_3D_global | RDisp_global | LDisp_global | X3DDisp_global | AreaTracking_global |
| --- | --- | --- | --- | --- | --- | --- | --- | --- | --- | --- | --- | --- |
| PC1_endo__end-systole_transported_size_and_shape_space | **0.5** | **-0.68** | **-0.66** | **0.45** | **0.26** | 0.17 | **0.23** | **0.57** | **0.66** | **0.65** | **0.79** | **-0.72** |
| PC2_endo__end-systole_transported_size_and_shape_space | 0.06 | **0.28** | **0.45** | **-0.25** | 0.15 | 0.01 | **0.25** | -0.02 | -0.08 | **-0.73** | **-0.51** | **0.34** |
| PC3_endo__end-systole_transported_size_and_shape_space | **-0.39** | -0.01 | **0.26** | 0.14 | **0.39** | **0.26** | **0.32** | **-0.4** | 0.02 | -0.15 | -0.03 | 0.09 |
| PC1_epi__end-systole_transported_size_and_shape_space | 0.13 | **-0.63** | **-0.65** | **0.38** | 0.23 | 0.17 | 0.18 | 0.23 | **0.54** | **0.73** | **0.75** | **-0.68** |
| PC2_epi__end-systole_transported_size_and_shape_space | **0.42** | 0.1 | 0.09 | -0.09 | **-0.25** | **-0.34** | **-0.24** | **0.37** | 0.03 | -0.09 | -0.06 | 0.1 |
| PC3_epi__end-systole_transported_size_and_shape_space | 0.23 | **-0.29** | **-0.25** | 0.11 | 0.2 | 0.12 | 0.11 | **0.27** | 0.19 | **0.45** | **0.39** | **-0.3** |
| PC1_endo__end-systole_transported_shape_space | **0.49** | **-0.62** | **-0.29** | **0.41** | **0.42** | **0.23** | **0.42** | **0.53** | **0.69** | **0.3** | **0.56** | **-0.57** |
| PC2_endo__end-systole_transported_shape_space | 0.07 | **0.32** | **0.35** | **-0.3** | 0.05 | -0.03 | 0.17 | 0 | -0.14 | **-0.68** | **-0.52** | **0.34** |
| PC3_endo__end-systole_transported_shape_space | **-0.39** | 0.1 | **0.29** | 0.04 | **0.39** | **0.25** | **0.35** | **-0.42** | -0.04 | **-0.29** | -0.15 | 0.18 |
| PC1_epi__end-systole_transported_shape_space | **0.33** | **-0.47** | **-0.39** | **0.26** | **0.27** | 0.14 | 0.21 | **0.4** | **0.48** | **0.52** | **0.58** | **-0.48** |
| PC2_epi__end-systole_transported_shape_space | **0.3** | 0.22 | 0.15 | -0.13 | **-0.38** | **-0.41** | **-0.32** | 0.23 | -0.09 | **-0.24** | -0.21 | 0.21 |
| PC3_epi__end-systole_transported_shape_space | **-0.25** | **0.24** | **0.26** | -0.08 | -0.08 | -0.03 | 0.01 | **-0.29** | -0.14 | **-0.47** | **-0.38** | **0.27** |
| PC1_endo_end-systole_size_and_shape_space | **0.25** | 0.01 | 0.02 | 0.05 | 0.02 | -0.16 | -0.02 | **0.26** | 0.11 | 0.21 | 0.22 | 0 |
| PC2_endo_end-systole_size_and_shape_space | **-0.28** | **0.51** | -0.08 | **-0.35** | -0.19 | -0.08 | -0.07 | **-0.26** | **-0.46** | -0.09 | **-0.36** | **0.39** |
| PC3_endo_end-systole_size_and_shape_space | -0.15 | 0.19 | **0.38** | -0.14 | -0.12 | -0.15 | -0.08 | -0.15 | -0.09 | **-0.45** | **-0.32** | 0.21 |
| PC1_epi_end-systole_size_and_shape_space | **0.29** | 0.18 | 0.1 | 0.01 | 0 | -0.19 | 0 | **0.24** | 0.01 | -0.01 | 0.03 | 0.18 |
| PC2_epi_end-systole_size_and_shape_space | -0.22 | **0.44** | 0.14 | **-0.29** | -0.19 | -0.12 | -0.04 | -0.27 | **-0.45** | **-0.45** | **-0.56** | **0.39** |
| PC3_epi_end-systole_size_and_shape_space | 0.16 | -0.07 | -0.07 | -0.06 | 0.22 | 0.13 | 0.2 | 0.17 | 0.17 | -0.13 | 0.03 | -0.09 |
| Shape_diff_endo_from_R_peak_transported_size_and_shape_space | **0.53** | **-0.7** | **-0.67** | **0.46** | **0.3** | 0.21 | **0.3** | **0.59** | **0.65** | **0.57** | **0.73** | **-0.74** |
| Shape_diff_epi_from_R_peak_transported_size_and_shape_space | 0.13 | **-0.63** | **-0.66** | **0.39** | 0.22 | 0.16 | 0.19 | **0.24** | **0.52** | **0.68** | **0.7** | **-0.69** |
| Shape_diff_endo_from_R_peak_transported_shape_space | **0.54** | **-0.52** | **-0.27** | **0.35** | **0.38** | 0.21 | 0.41 | **0.56** | **0.62** | 0.13 | **0.42** | **-0.48** |
| Shape_diff_epi_from_R_peak_transported_shape_space | **0.42** | **-0.34** | **-0.3** | **0.25** | 0.13 | 0 | 0.14 | **0.49** | **0.45** | **0.26** | **0.42** | **-0.36** |
| Shape_diff_endo_from_R_peak_size_and_shape_space | **0.53** | **-0.67** | **-0.64** | **0.44** | **0.28** | 0.19 | **0.29** | **0.59** | **0.64** | **0.54** | **0.71** | **-0.71** |
| Shape_diff_epi_from_R_peak_size_and_shape_space | 0.18 | **-0.59** | **-0.64** | **0.36** | 0.21 | 0.15 | 0.2 | **0.28** | **0.52** | **0.62** | **0.68** | **-0.65** |
| PC1_traj_shape_endo_size_and_shape_space | **0.55** | **-0.65** | **-0.66** | **0.42** | **0.26** | 0.17 | **0.25** | **0.62** | **0.66** | **0.6** | **0.77** | **-0.7** |
| PC2_traj_shape_endo_size_and_shape_space | 0.02 | -0.01 | 0.12 | 0.19 | 0.06 | 0.03 | 0.05 | 0.05 | 0.13 | -0.16 | -0.01 | 0 |
| PC3_traj_shape_endo_size_and_shape_space | -0.01 | **-0.31** | **-0.24** | 0.15 | 0.12 | 0.17 | 0.16 | 0.01 | 0.15 | 0.05 | 0.07 | **-0.32** |
| PC1_traj_shape_epi__size_and_shape_space | 0.17 | **-0.61** | **-0.69** | **0.36** | 0.21 | 0.14 | 0.18 | **0.28** | **0.55** | **0.7** | **0.75** | **-0.68** |
| PC2_traj_shape_epi_size_and_shape_space | 0.08 | 0.06 | **0.24** | 0.19 | 0.03 | -0.08 | -0.04 | 0.07 | 0.11 | -0.11 | 0.02 | 0.1 |
| PC3_traj_shape_epi_size_and_shape_space | -0.19 | -0.14 | -0.02 | 0.1 | 0.1 | 0.15 | 0.1 | -0.16 | 0 | 0.01 | -0.05 | -0.13 |
| PC1_traj_shape_endo_shape_space | **0.59** | **-0.52** | **-0.27** | **0.34** | **0.38** | 0.18 | **0.39** | **0.61** | **0.67** | 0.17 | **0.49** | **-0.48** |
| PC2_traj_shape_endo_shape_space | 0.03 | -0.13 | -0.2 | 0.2 | -0.09 | -0.02 | -0.11 | 0.11 | 0.16 | 0.09 | 0.12 | -0.18 |
| PC3_traj_shape_endo_shape_space | **0.25** | 0.15 | 0.04 | -0.06 | -0.09 | -0.22 | -0.15 | 0.19 | 0.06 | -0.08 | 0.01 | 0.15 |
| PC1_traj_shape_epi_shape_space | **-0.4** | **0.29** | **0.32** | -0.22 | -0.16 | -0.05 | -0.16 | **-0.47** | **-0.4** | **-0.3** | **-0.43** | **0.33** |
| PC2_traj_shape_epi_shape_space | **0.25** | -0.06 | 0 | 0.1 | -0.23 | -0.17 | **-0.27** | 0.19 | 0.2 | -0.07 | 0.05 | -0.02 |
| PC3_traj_shape_epi_shape_space | -0.03 | 0.07 | 0.07 | -0.04 | -0.01 | -0.04 | 0.01 | -0.06 | -0.03 | -0.01 | -0.03 | 0.1 |
| size_traj_shape_endo_size_and_shape_space | **0.54** | **-0.68** | **-0.67** | **0.44** | **0.27** | 0.19 | **0.26** | **0.61** | **0.68** | **0.59** | **0.77** | **-0.72** |
| size_traj_shape_epi_size_and_shape_space | 0.15 | **-0.61** | **-0.69** | **0.36** | 0.22 | 0.15 | 0.19 | **0.26** | **0.54** | **0.69** | **0.74** | **-0.68** |
| size_traj_shape_endo__shape_space | **0.57** | **-0.52** | **-0.24** | **0.33** | 0.37 | 0.18 | **0.38** | 0.59 | 0.67 | 0.15 | 0.47 | -0.48 |
| size_traj_shape_epi_shape_space | **0.37** | **-0.29** | **-0.34** | **0.21** | 0.17 | 0.07 | 0.17 | **0.45** | **0.39** | **0.3** | **0.43** | **-0.33** |
| PC1-PC2_angle_endo_size_and_shape_space | 0.09 | **0.28** | **0.45** | **-0.25** | 0.12 | -0.04 | 0.23 | 0.01 | -0.04 | **-0.72** | **-0.48** | **0.35** |
| PC1-PC3_angle_endo_size_and_shape_space | **0.41** | 0.05 | 0.03 | -0.03 | -0.18 | **-0.31** | -0.17 | **0.35** | 0.05 | -0.01 | 0.02 | 0.04 |
| PC1-PC2_angle_epi_size_and_shape_space | 0.1 | **0.3** | **0.33** | **-0.28** | 0.04 | -0.09 | 0.15 | 0.03 | -0.08 | **-0.65** | **-0.46** | **0.33** |
| PC1-PC3_angle_epi_size_and_shape_space | **0.33** | 0.02 | 0.03 | 0.05 | -0.18 | **-0.28** | -0.14 | **0.26** | 0.07 | -0.03 | 0.01 | 0.02 |
| PC1-PC2_angle_endo_shape_space | 0.09 | **0.28** | **0.45** | **-0.25** | 0.12 | -0.04 | 0.23 | 0.01 | -0.04 | **-0.72** | **-0.48** | **0.35** |
| PC1-PC3_angle_endo_shape_space | **0.41** | 0.05 | 0.03 | -0.03 | -0.18 | **-0.31** | -0.17 | **0.35** | 0.05 | -0.01 | 0.02 | 0.04 |
| PC1-PC2_angle_epi_shape_space | 0.1 | **0.3** | **0.33** | **-0.28** | 0.04 | -0.09 | 0.15 | 0.03 | -0.08 | **-0.65** | **-0.46** | **0.33** |
| PC1-PC3_angle_epi_shape_space | **0.33** | 0.02 | 0.03 | 0.05 | -0.18 | **-0.28** | -0.14 | **0.26** | 0.07 | -0.03 | 0.01 | 0.02 |
| RV_size_and_shape_space | -0.01 | -0.17 | **-0.32** | 0.22 | 0.11 | 0.1 | 0.05 | 0 | 0.18 | **0.43** | **0.34** | -0.2 |
| RV_shape_space | **0.33** | -0.17 | **-0.3** | 0.07 | 0.02 | 0 | 0.02 | **0.32** | **0.38** | 0.2 | **0.36** | -0.2 |

Representation of matrix above (rows and columns match) via colour code indicating the direction (blue = positive; red = negative) of correlation and its strength symbolized by circle size. Empty cells indicate non significant relationships.
